# Understanding β-strand mediated protein–protein interactions: tuning binding behaviour of intrinsically disordered sequences by backbone modification†

**DOI:** 10.1039/d4sc02240h

**Published:** 2024-06-06

**Authors:** Emma E. Cawood, Emily Baker, Thomas A. Edwards, Derek N. Woolfson, Theodoros K. Karamanos, Andrew J. Wilson

**Affiliations:** a Astbury Centre for Structural Molecular Biology, University of Leeds Woodhouse Lane Leeds LS2 9JT UK a.j.wilson.1@bham.ac.uk; b School of Chemistry, University of Leeds Woodhouse Lane Leeds LS2 9JT UK; c School of Biochemistry, University of Bristol Medical Sciences Building, University Walk Bristol BS8 1TD UK d.n.woolfson@bristol.ac.uk; d BrisSynBio, University of Bristol Life Sciences Building, Tyndall Avenue Bristol BS8 1TQ UK; e School of Molecular and Cellular Biology, University of Leeds Woodhouse Lane Leeds LS2 9JT UK; f School of Chemistry, University of Bristol Cantock's Close Bristol BS8 1TS UK; g Department of Life Sciences, Imperial College London London SW7 2BX UK t.karamanos@imperial.ac.uk; h School of Chemistry, University of Birmingham Edgbaston Birmingham B15 2TT UK; i College of Biomedical Sciences, Larkin University 18301 N Miami Ave #1 Miami FL 33169 USA

## Abstract

A significant challenge in chemical biology is to understand and modulate protein–protein interactions (PPIs). Given that many PPIs involve a folded protein domain and a peptide sequence that is intrinsically disordered in isolation, peptides represent powerful tools to understand PPIs. Using the interaction between small ubiquitin-like modifier (SUMO) and SUMO-interacting motifs (SIMs), here we show that *N*-methylation of the peptide backbone can effectively restrict accessible peptide conformations, predisposing them for protein recognition. Backbone *N*-methylation in appropriate locations results in faster target binding, and thus higher affinity, as shown by relaxation-based NMR experiments and computational analysis. We show that such higher affinities occur as a consequence of an increase in the energy of the unbound state, and a reduction in the entropic contribution to the binding and activation energies. Thus, backbone *N*-methylation may represent a useful modification within the peptidomimetic toolbox to probe β-strand mediated interactions.

## Abstract 2

Systematic backbone *N*-methylation of a SIM peptide results in faster, tighter binging to SUMO driven by a reduced entropy of activation; this opens new opportunities to develop inhibitors of β-strand mediated protein–protein interactions.

## Graphical abstract

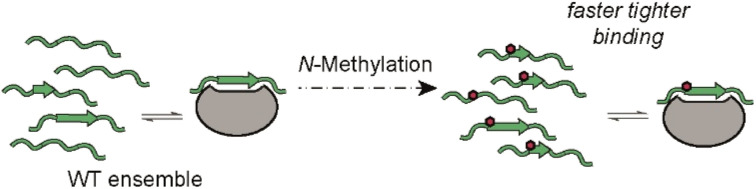

## Introduction

Protein–protein interactions (PPIs) drive and regulate many biological functions.^1–3^ Understanding the molecular mechanism by which protein–protein interactions occur is central to understanding function.^4^ Such mechanistic understanding can support development of tools that can modulate PPIs to act as probes of healthy and disease states of biological processes, and prime drug discovery efforts.^5–8^ Many PPIs employ short peptides for recognition;^9,10^ aside from serving as templates for designing PPI inhibitors,^11–16^ a feature of short recognition peptides is that they are often intrinsically disordered^17^ and undergo disorder-order transitions, *e.g.*, to form α-helices^18^ or β-strands^19^ on PPI formation. The association binding kinetics of coupled folding and binding are influenced by the larger hydrodynamic radius, flexibility and folding propensity of intrinsically disordered regions/proteins (IDR/Ps) in comparison to folded proteins, alongside the abundance of exposed charged residues which are often associated with encounter complex formation.^17,20^ IDR/Ps in their apo form can also populate a bound-folded state to support a conformational selection binding mechanism. Whilst IDR/Ps that populate a helical bound conformation can be easily evidenced by NMR chemical shifts, β-strand conformations are more challenging to characterise; a propensity to form extended structures is normally captured by NMR, but the extended ensemble typically contains numerous conformations, only some of which are binding-competent. The synthetic accessibility of peptides makes them ideal tools to study biomolecular mechanisms; modifications that alter the conformational landscape of a disordered peptide can be used to bias the energy landscape to probe binding mechanisms in a systematic way.^21,22^

*N*-Methylation of backbone amides has been shown to improve the affinity, interaction specificity, solubility, membrane permeability, and proteolytic stability of peptides.^23,24^ However, these studies have generally focused on cyclic peptides. There are far fewer reports on *N*-methylation of linear peptides, and even fewer for peptides solely composed of l-amino acids.^25–28^*N*-Methylation can restrict the conformations accessible to a peptide, as it disfavours backbone conformations in the bottom-left quadrant of the Ramachandran plot, which includes the α-helical region, *ϕ* ≈ −60° and *ψ* ≈ −50°.^29,30^ However, it also allows access to alternative conformations by lowering the difference in stability between *cis*/*trans* amide rotamers.^31,32^ Nonetheless, the precise manner in which *N*-methylation can be used to alter the backbone conformational preferences of linear peptides, in particular understanding what determines the extent to which *N*-methylation favours more-extended structures, is less explored. *N*-Methylation also changes the hydrogen-bonding capabilities of peptides. In turn, this could alter the ability of a peptide to bind to protein targets, but it can also improve the physical properties of peptides; for instance, reducing propensities to self-assemble into amyloid-like structures,^33,34^ and susceptibilities to certain proteases.^35^ Thus, in this work, we sought to explore the extent to which backbone *N*-methylation might serve as a tool to inform on and modulate IDR/P binding mechanism. Using the interaction between small ubiquitin-like modifier (SUMO) and SUMO-interacting motifs (SIMs) as a model to explore the effects of peptide *N*-methylation, we show that whilst backbone modification of some sites abrogates binding, at others it increases the peptide–protein association rate (*k*_on_) resulting in small increases in binding affinity. For the latter, this behaviour can be rationalized as follows: *N*-methylation restricts the accessible peptide conformations, in effect predisposing them for target recognition. This is achieved by raising the energy of the unbound state and decreasing the activation energy (entropy) required for binding.

## Results

### Position-dependent effects of *N*-methylation on binding affinity

**Fig. 1:**
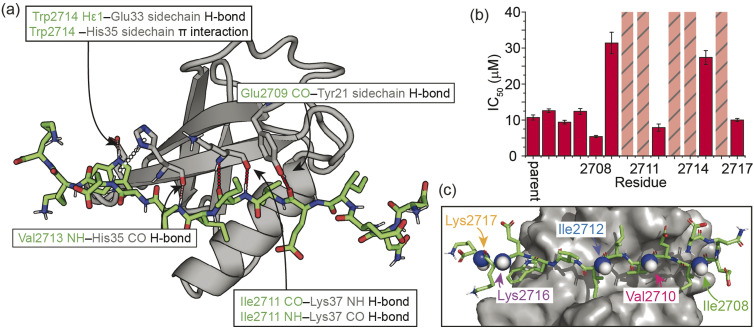
Overview of the interaction between the M-IR2 region of RanBP2 (SIM_2705–2717_) and *h*SUMO-1_18–97_ (SUMO), and the effects of backbone modification on the strength of interaction: (a) lowest energy structure of the NMR-derived structural ensemble for SIM/SUMO (PDB ID, 2LAS), highlighting key interactions (identified using Arpeggio)^36^ between the parent SIM peptide and SUMO; (b) differences in potency for variant SIM peptides as determined in fluorescence anisotropy competition assays; hatched bars highlight variants for which precise IC_50_ values could not be measured; see ESI Fig. S1† for conditions and titration data; (c) sites of *N*-methylation for which detailed NMR analyses were performed (Fig. 2–5).

As a model β-strand-mediated PPI to explore the effect of *N*-methylation on peptide conformation and protein binding, we chose the interaction between human SUMO-1_18–97_ (SUMO) and the SIM motif found in the M-IR2 region of RanBP2. Our chosen SIM sequence – Ac-Asp-Asn-Glu-Ile-Glu-Val-Ile-Ile-Val-Trp-Glu-Lys-Lys-NH_2_ (herein referred to as the ‘parent SIM peptide’) – was taken from Namanja *et al.*,^37^ who modified the wild-type RanBP2 M-IR2 SIM_2705–2717_ sequence to make it more amenable to biophysical study. This SIM peptide is intrinsically disordered in the absence of a binding partner,^38^ but binds to SUMO through β-augmentation with low micromolar affinity.^37^ Key non-covalent interactions present in the bound complex (Fig. 1a) include hydrogen bonds from SUMO to the backbone of SIM residues Glu2709, Ile2711, and Val2713, and side chain hydrogen bonds and π interactions to SIM Trp2714. Hydrophobic packing of isoleucine and valine side chains from SIM along the SUMO binding cleft also contributes to binding affinity; previously, we conducted an alanine scan on this sequence and identified a contiguous stretch of hot-spot residues from Val2710 to Trp2714 (VIIVW; ΔΔ*G* ≥ 4.2 kJ mol^−1^).^38^ This corresponds to the (V/I)X(V/I)(V/I) or (V/I)(V/I)X(V/I/L) consensus motif commonly found in SIMs.^37,39^ In addition, Glu2709 was just below the threshold for being classified as a hot-spot residue.

On this basis, we performed a systematic backbone *N*-methyl scan for all 13 residues of the parent SIM peptide. Peptides were prepared using Fmoc-based solid phase peptide synthesis (see the ESI† for procedures and characterization) and their relative binding affinities to SUMO were estimated using a fluorescence anisotropy competition assay to allow prioritization of sequences for more detailed study by NMR (incl. accurate affinity determination).^38^ Changes in inhibitory potency were observed both within and outside the consensus hot-spot region. *N*-Methylation at six sites (Me-Asp2705, Me-Asn2706, Me-Glu2707, Me-Ile2708, Me-Ile2712, Me-Lys2717) led to similar or slightly improved inhibitory potency to the parent SIM peptide (Fig. 1b and S1, Table S1†). By contrast, *N*-methylation at five sites (Me-Val2710, Me-Ile2711, Me-Ile2713, Me-Trp2714, and Me-Lys2716) led to significantly diminished inhibitory potency, and a minor reduction in potency at the two remaining sites (Me-Ile2709, and Me-Glu2715).

### Effects of *N*-methylation on the unbound peptide

We next sought to rationalise the changes in potency observed as a consequence of *N*-methylation. For some variants (*e.g.*, Me-Ile2711 and Me-Val2713), disruption of binding could reasonably be attributed to loss of key backbone hydrogen bonds between SIM and SUMO, and/or the steric hindrance conferred by the methyl group disrupting adjacent interactions (Fig. 1a). For the remaining variants however, the observed changes in IC_50_ could not be readily explained based on changes to hydrogen bonding interactions or possible steric clashes between the variant peptide and the protein. We therefore considered that changes in binding affinity may instead arise from changes in backbone conformation (which can in turn affect side-chain conformations) or changes in the electron density of amide bonds. With a selection of peptides from across the *N*-methylated SIM series (Fig. 1c), this was investigated using solution NMR methods and computational analyses for the unbound peptides.

Three *N*-methylated SIM variants with IC_50_ values close to that of the parent SIM peptide (Me-Ile2708, Me-Ile2712, Me-Lys2717) and two variants with less potent IC_50_ values (Me-Val2710 and Me-Lys216) were selected (Fig. 1c) to interrogate the determinants of binding affinity, relative to the parent SIM peptide. Using one-dimensional ^1^H NMR experiments, we ruled out changes in the population of the *cis* isomer at the *N*-methylated amide bond and changes in peptide oligomeric state as drivers of the observed changes in potency (Fig. S2 and S3†).

**Fig. 2:**
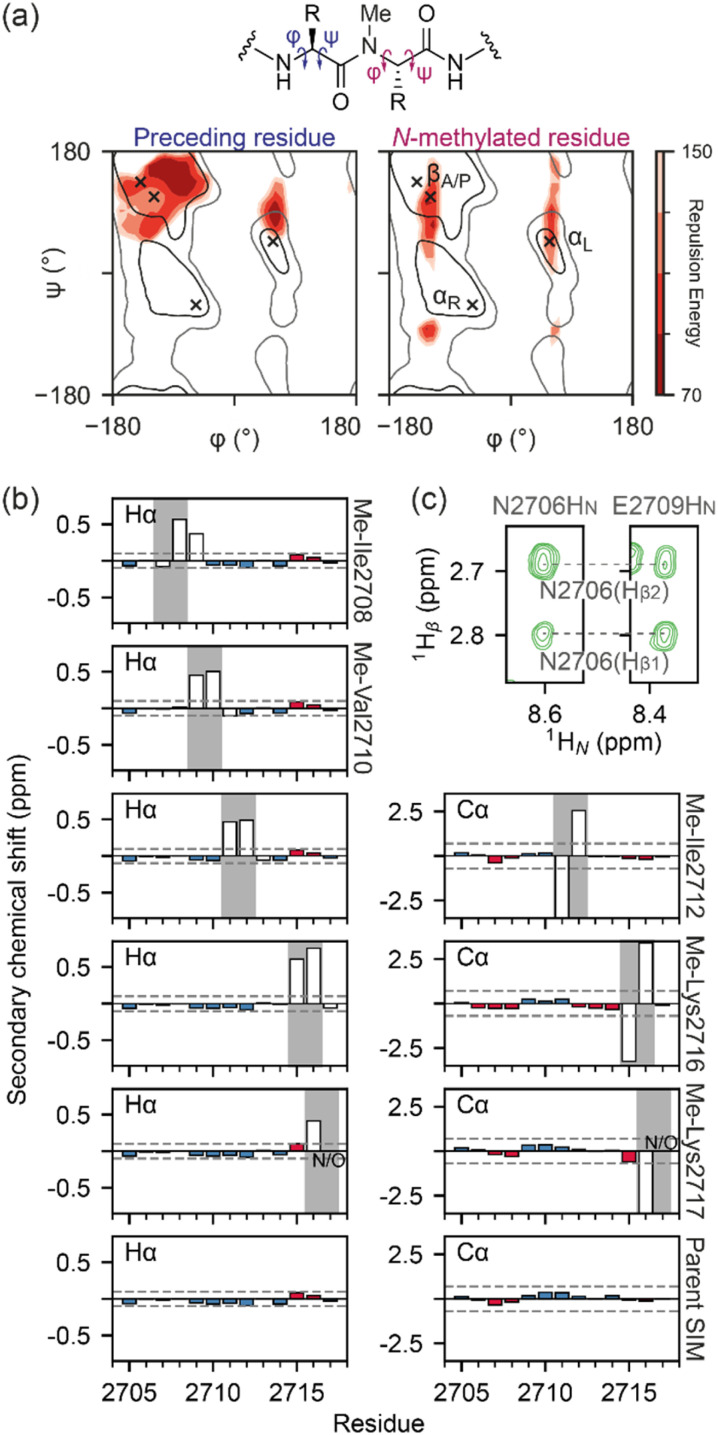
Conformational analysis of *N*-methylated peptides: (a) Ramachandran plots of an *N*-methylated residue (*i*) and the preceding residue (*i*-1) in a peptide, as a function of the repulsion energy as calculated by XPLOR-NIH (see ESI Materials and Methods†); (b) Hα or Cα secondary chemical shifts for SIM and *N*-methylated variant peptides. Propensity for β-strand/α-helix is shown by red/blue bars respectively with threshold for significant propensity denoted by dashed grey line. Chemical shift values around the methylation site are shown as open bars; (c) ^1^H–^1^H NOESY strips of the Me-Ile2708 variant peptide (5 °C, 500 μM peptide, 20 mM sodium phosphate, pH 7.4, 0.02% NaN_3_, mixing time 500 ms) highlighting the E2706-H_β_ to E2709-H_N_ NOE.

Simulated annealing calculations of *N*-methylated peptides indicated that methylation of backbone amide bonds could restrict the accessible *phi* (*ϕ*) and *psi* (*ψ*) angles, rendering the α-helical region of Ramachandran space inaccessible (Fig. 2a). As the parent SIM sequence has been shown to lack stable secondary structure in the absence of a binding partner,^38^ it is possible that some of the differences in binding affinity observed could be explained by changes to the secondary structure propensity of *N*-methylated SIM variants. NMR chemical shifts of backbone nuclei (Hα, Cα, and Cβ) can be used as reporters of even small changes in secondary structure of disordered proteins/peptides,^40^ and α-like or β-like chemical shifts are indicative of an increase in the population of those secondary structures. Therefore, the backbone and side chain chemical shifts of parent SIM and its *N*-methylated variants were assigned using ^1^H–^1^H TOCSY, ^1^H–^1^H NOESY spectra, and natural abundance ^1^H–^13^C HSQC spectra. As anticipated, the backbone chemical shifts of the parent SIM were consistent with those for a fully unstructured (random coil) peptide (Fig. S4†). Comparison of chemical shifts within the *N*-methylated region of each variant, relative to parent SIM, is complicated by the fact that *N*-methylation will increase the electron density of the associated amide bond, due to the electron-donating character of the methyl group. In the absence of any structural changes, we expect the chemical shift of neighbouring H_A_ atoms (*i.e.*, H_A_ of the *N*-methylated residue and H_A_ of the preceding residue) to be shifted downfield when compared to parent SIM chemical shifts. This is what is observed for Me-Val2710, Me-Ile2712, Me-Lys2716, and Me-Lys2717 (Fig. 2b and S5†). Excluding atoms whose chemical environment is directly impacted by the introduction of the *N*-methyl group (*i.e.*, atoms within six bonds of the methyl carbon), the measured backbone chemical shifts of all five *N*-methylated variants differed very little from the shifts of the parent SIM, indicating that no long-range secondary structural elements (*e.g.*, extended, β-rich structure) had been detected for these unbound peptides. In further support of this conclusion, we observed no NOE's indicative of helical or strand conformations.

For Me-Ile2708, the H_A_ chemical shift pattern surrounding the *N*-methylated peptide bond differs for both the *trans* and *cis* isomers; the residue before the *N*-methylated amide is significantly more upfield than in the other SIM variants, while the residue after the *N*-methylated amide is more downfield (Fig. 2b), suggesting the possibility of a turn-like collapse.^41,42^ An NOE between N2706-H_A_ and E2709-H_N_ was also observed for the *trans* isomer of Me-Ile2708 (Fig. 2c), indicating some localized structuring of residues at the *N* terminus of this variant. However, we note that the *N* terminus does not participate in SIM/SUMO recognition and thus it is difficult to predict how such ordering will affect binding.

Overall, our NMR analysis on the unbound peptides strongly indicates that restriction of the available conformational space as a consequence of *N*-methylation does not induce significant changes in secondary structure. Thus, the observed changes in affinity likely arise from altered binding kinetics or differences in the bound SIM/SUMO structure.

### Binding kinetics of the SIM/SUMO interaction can be derived from relaxation-based NMR measurements

**Fig. 3:**
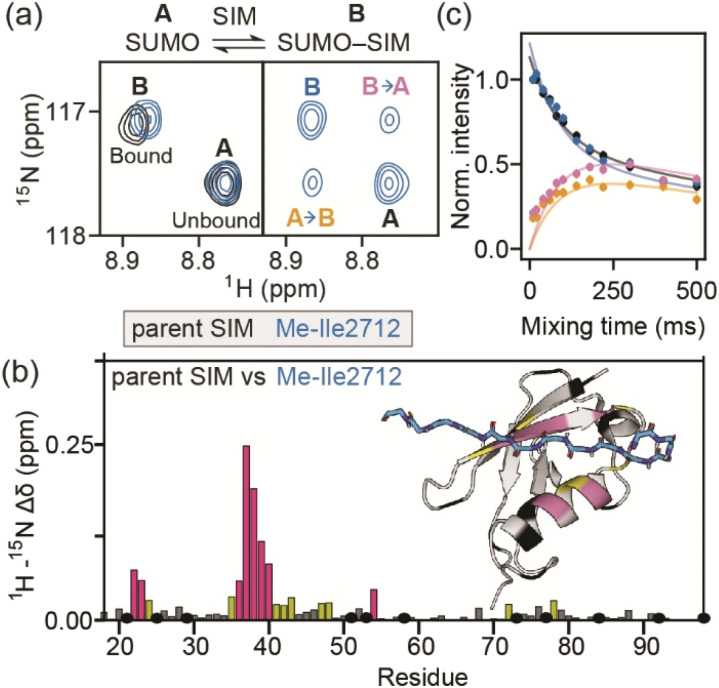
(a) ^1^H–^15^N HSQC spectra of ^15^N-SUMO bound to parent SIM (black) or Me-Ile2712 (blue). Two sets of resonances corresponding to unbound (state A) and bound (state B) SUMO are observed. The same spectral region in the ^15^N ZZ-exchange experiment with 100 ms mixing time is shown for Me-Ile2712. Exchange cross-peaks describing the flow of magnetization from state A to B (and *vice versa*) are labelled accordingly. (b) Combined ^1^H–^15^N chemical shift differences for SUMO residues when in complex with the parent SIM *vs.* when in complex with the Me-Ile2712 SIM variant. SUMO residues which could not be confidently assigned are shown in black, while the remaining residues are coloured according to the magnitude of the chemical shift difference, relative to the standard deviation (*σ*) of the dataset (<1*σ*, grey; 1–2*σ*, yellow; ≥2*σ*, pink; a cartoon representation of the SIM/SUMO complex coloured using the same colour scheme is shown on the right). (c) Measured intensities (dots) for the auto and cross-peaks from the ^15^N ZZ-exchange experiment for Me-Ile2712 shown in (a) as a function of mixing time. Solid lines represent fits to the simple 2-state model shown in (a).

**Fig. 4:**
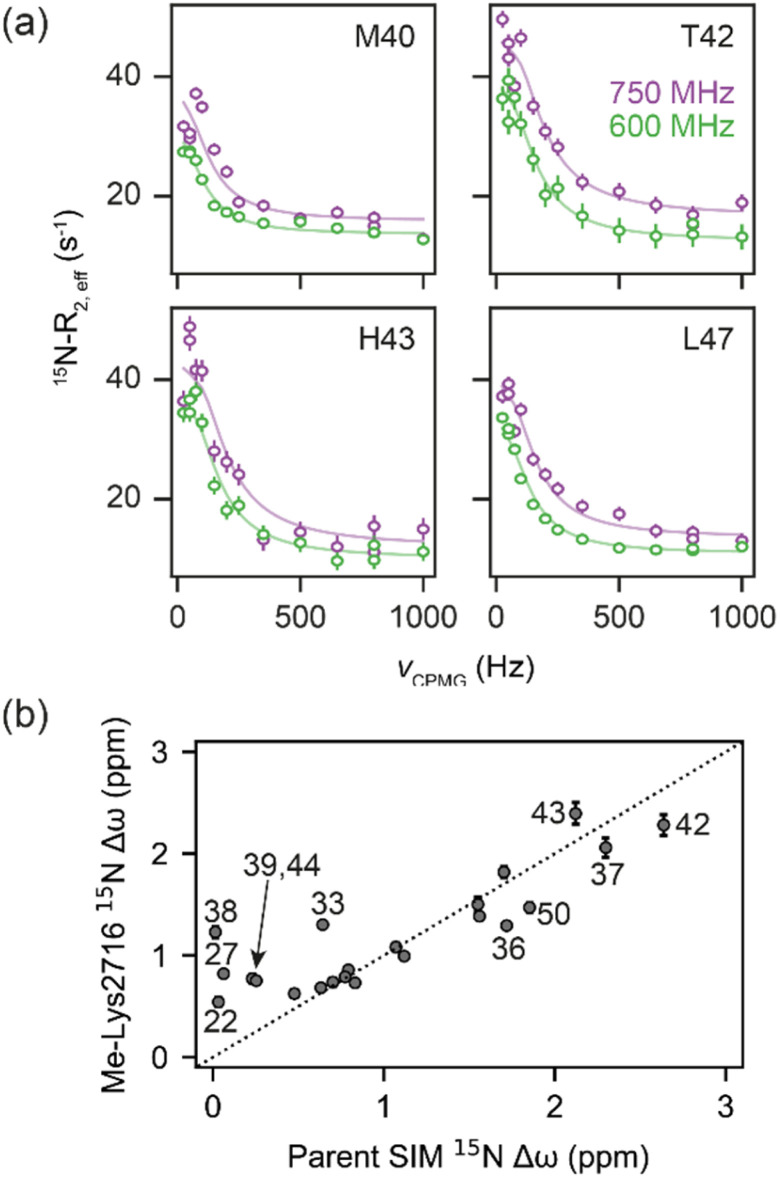
(a) ^15^N CPMG profiles of ^15^N-SUMO in the presence of Me-Lys2716 at 750 MHz (purple) and 600 MHz (green). Experimental data are shown as dots, while fits to a 2-state model (see Fig. 3a) are shown as solid lines. (b) Correlation of fitted ^15^N Δ*ω* values for the Me-Lys2716 bound SUMO with those experimentally observed for SUMO bound to parent SIM (Pearson correlation coefficient ∼ 0.86).

**Fig. 5:**
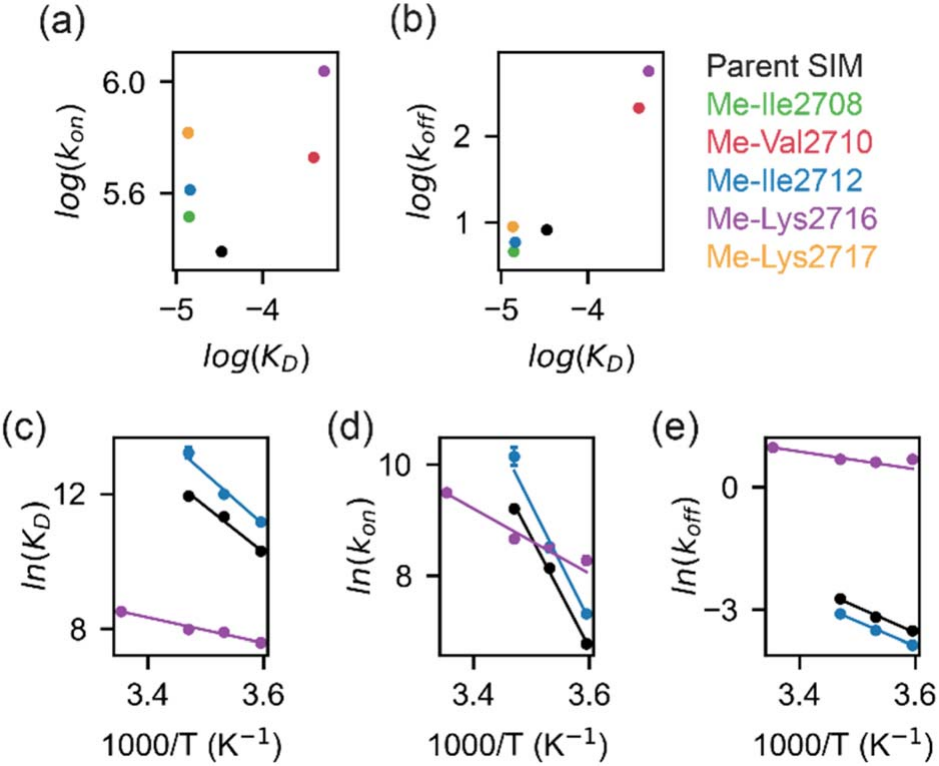
(a) Linear free energy relationship (LFER) plot depicting relationship between *k*_on_ and *K*_D_ for the binding of parent SIM (black), Me-Ile2708 (green), Me-Val2710 (red), Me-Ile2712 (blue), Me-Lys2716 (purple) or Me-Lys2717 (orange) to SUMO. (b) LFER plot depicting relationship between *k*_off_ and *K*_D_ for binding of SIM variants (colours as for (a)) to SUMO. (c) Van't Hoff plots of the temperature-dependence of binding affinities for parent SIM (black), Me-Ile2712 (blue) and Me-Lys2716 (purple); (d and e) Eyring plots for the association rate (d), *k*_on_, and dissociation rate (e), *k*_off_, for parent SIM (black), Me-Ile2712 (blue) and Me-Lys2716 (purple). For (c)–(e), experimental data-points are shown as dots and solid lines represent linear fits to the data.

To determine more accurately the thermodynamic parameters for binding and investigate the binding kinetics of the parent SIM and its *N*-methylated variant peptides, NMR-relaxation based methods were used (Fig. 3 and 4). Initially, a series of ^1^H–^15^H NMR spectra were acquired using ^15^N-enriched SUMO in the presence of natural abundance ^14^N-SIM peptides. For the parent SIM peptide and for variants with similar values of Δ*G*_bind_ (Me-Ile2708, Me-Ile2712, Me-Lys2717), peptide binding to SUMO was observed in the slow exchange regime on the chemical shift timescale, giving rise to two sets of ^1^H–^15^N resonances for residues in the SIM-binding pocket (helix α1 and strand β2 of SUMO), corresponding to the bound and unbound species (Fig. 3a). For these tighter-binding variants, bound-state chemical shift differences relative to SUMO/parent SIM (Δ*ω*) were only observed for residues expected to lie in proximity to the *N*-methylated SIM residue (Fig. 3b, S6 and S7†),^37^ indicating that these peptides bind SUMO in the canonical binding conformation. Non-overlapping exchange cross-peaks were evident for a subset of residues when the ^15^N magnetizations of the bound and unbound states were mixed following t1 (^15^N) evolution (ZZ-exchange spectroscopy). Global fitting of the intensities of auto and cross peaks as a function of mixing time to McConnel equations (Fig. 3c, S8–S11; ESI† for methods) gave apparent first-order association *k*^app^_on_ and first-order dissociation *k*_off_ rates for binding, which were converted to *k*_on_ and dissociation equilibrium constants (*K*_D_). For the parent, Me-Ile2708, Me-Ile2712, and Me-Lys2717 peptides, ^15^N ZZ-exchange data at 5 °C fit well to a 2-state bound-unbound model, yielding *K*_D_ values in the low micromolar range (Table 1). These confirm an increase in affinity for the *N*-methylated series (*e.g.*, ΔΔ*G*_Me-Ile2712_*vs.*_parent_ ∼ 2 kJ mol^−1^). For this set of four peptides, *k*_off_ values were similar (4–8 s^−1^), suggesting that *k*_on_ is the primary cause of changes in *K*_D_. Indeed, a positive correlation was observed between these two parameters (see Fig. 5a). The observed faster association rates, in the absence of any changes in the charge of the variant SIM peptides, could suggest that *N*-methylation at these locations conformationally predisposes these peptides for SUMO binding.

**Table 1:** Binding affinities and rates for the binding of *N*-methylated SIM peptides to SUMO. *K*_D_, *k*_on_, and *k*_off_ values were determined for select SIM variants by NMR (using ^15^N ZZ-exchange experiments for tighter binding variants, and CPMG experiments for weaker binding variants). NMR data was acquired in 20 mM sodium phosphate, pH 7.4, 150 mM NaCl, 2 mM DTT, 0.02% NaN_3_, 5 °C

Peptide	*K*_D_ at 5 °C (μM)	*k*_on_ (s^−1^ M^−1^) × 10^5^	*k*_off_ (s^−1^)
Me-Ile2708	14.0 ± 0.4	3.27 ± 0.09	4.58 ± 0.04
Me-Val2710	388 ± 7	5.36 ± 0.10	208 ± 1
Me-Ile2712	14.5 ± 0.9	4.10 ± 0.28	5.90 ± 0.08
Me-Lys2716	510 ± 40	10.9 ± 0.7	553 ± 17
Me-Lys-2717	13.6 ± 0.5	6.57 ± 0.27	8.95 ± 0.12
Parent SIM	33.6 ± 0.9	2.45 ± 0.07	8.22 ± 0.08

For the weak peptide binders (Me-Val2710 and Me-Lys2716), only the unbound SUMO resonances were observed in ^1^H–^15^N HSQC spectra, even at high SIM concentrations. This suggests that the population of the bound complex is small and/or that binding does not take place in the slow chemical shift timescale. Thus, to investigate the binding kinetics of those variants, we used Carr–Purcell–Meiboom–Gill (CPMG) relaxation dispersion that is sensitive to exchange between states with skewed populations on the millisecond timescale.^43^ Large CPMG profiles for SUMO residues in the SIM-binding site were observed at 5 °C upon addition of Me-Val2710 or Me-Lys2716 (Fig. 4a and S12–S14†). These profiles were absent for apo SUMO (Fig. S15†), suggesting that the observed millisecond dynamics are due to transient SIM binding. Global fitting of CPMG data at 600 and 750 MHz to a 2-state model yielded excellent fits (Fig. 4, S12 and S13†). CPMG-derived chemical shifts of the transiently populated, bound state of SUMO in the presence of Me-Val2710 or Me-Lys2716 correlated well with those of the stable, parent SIM-bound state, with the exception of some residues in the SIM binding pocket (see Fig. S12b and S13b†), suggesting that the bound state of SUMO in the presence of these SIM variants is not significantly different to that with parent SIM. However, *k*_off_ values or the weakly bound variants increased by more than 50-fold (∼200–550 s^−1^; Fig. 5b) – therefore, while the structure of SUMO in the bound state complex is unchanged by *N*-methylation, *N*-methylation at residues Val2710 and Lys2716 either prevents or hinders the ability of the SIM peptide to adopt its canonical binding conformation, leading to fast dissociation and a lower affinity interaction. It is surprising that Me-Val2710 does not show improved binding affinity as the amide NH is not involved in hydrogen-bonding and methylation should restrict Ramachandran space to conformations that favour binding.^44^ Therefore, it is likely that methylation adversely influences the accessible *χ* space of the isopropyl side chain of Val2710 (a hot-spot residue), making it incompatible with SUMO binding. In the case of Lys2716, we consider it more likely that methylation of this residue perturbs the allowable *χ* space for Trp2714.

All together, our results suggest that the restriction of the conformational space available to *N*-methylated peptides can increase association kinetics which correlates with enhanced binding. Conversely, in some cases, if the modified peptide cannot adopt a stable binding conformation, *N*-methylation can favour dissociation which correlates with diminished binding.

### Increased affinity is due to a lower association activation barrier

To determine thermodynamic parameters for the SIM/SUMO interaction, we studied the temperature dependence of the NMR-derived exchange parameters^45^ for the parent peptide, along with those for a strong and a weak binding *N*-Me variant – Me-Ile2712 and Me-Lys2716, respectively (Fig. S8, S9, and S14†). Van't Hoff analysis of the calculated binding affinities revealed SIM binding to be an entropically-driven process (Table 2 and Fig. 5c). In comparison to the parent SIM peptide, the entropy of Me-Ile2712 binding at 25 °C (*T*Δ*S*_bind_) increased by ∼15 kJ mol^−1^, which was only partially compensated by the ∼12 kJ mol^−1^ increase in enthalpy (Δ*H*_bind_) (Fig. 5c, Table 2), leading to a more favourable free energy of binding (Δ*G*_bind_). Thus, the increased affinity for Me-Ile2712, relative to the parent, is entropy-driven. For Me-Lys2716, both Δ*H*_bind_ and *T*Δ*S*_bind_ were diminished, resulting in a smaller Δ*G*_bind_ and a weak affinity.

**Table 2:** Binding thermodynamics for the SIM/SUMO interaction at 25 °C. Values were calculated by fitting the Van't Hoff or Eyring equations. Uncertainties represent the standard deviation of the fitted parameters, calculated in a Monte-Carlo fashion

	Δ*H* (kJ mol^−1^)	*T*Δ*S* (kJ mol^−1^)	Δ*G* (kJ mol^−1^)
**Parent SIM**			
Binding (*K*_D_)	113.1 ± 0.7	146.7 ± 2.8	−33.6 ± 1.0
Association (activation, *k*_on_)	164.8 ± 1.0	134.6 ± 1.0	30.2 ± 1.2
Dissociation (activation, *k*_off_)	52.0 ± 0.3	−11.9 ± 0.3	64.0 ± 0.4

**Me-Ile2712**			
Binding (*K*_D_)	125.2 ± 0.2	161.8 ± 2.0	−36.6 ± 2.0
Association (activation, *k*_on_)	172.5 ± 2.1	145.5 ± 2.1	27.5 ± 2.2
Dissociation (activation, *k*_off_)	50.2 ± 0.3	−14.7 ± 0.2	64.8 ± 0.4

**Me-Lys2716**			
Binding (*K*_D_)	31.4 ± 0.2	52.5 ± 0.2	−21.1 ± 0.3
Association (activation, *k*_on_)	48.5 ± 0.2	13.0 ± 0.2	35.4 ± 0.3
Dissociation (activation, *k*_off_)	17.9 ± 0.1	−38.5 ± 0.1	56.5 ± 0.1

Eyring plots were obtained for the temperature-dependence of the *k*_on_ and *k*_off_ rates (Fig. 5d and e), from which the association/dissociation activation enthalpies (Δ*H*^‡^_ass_/Δ*H*^‡^_diss_), activation/dissociation entropies (*T*Δ*S*^‡^_ass_/*T*Δ*S*^‡^_diss_), and thus activation/dissociation free energies (Δ*G*^‡^_ass_/Δ*G*^‡^_diss_), could be determined. The association of SIM and its variants with SUMO has an enthalpic activation barrier in all cases (Table 2), while peptide dissociation has an enthalpic barrier for parent SIM and Me-Ile2712, and a smaller entropic barrier for Me-Lys2716 (Fig. 5d, Table 2). At 25 °C, *T*Δ*S*^‡^_ass_ for Me-Ile2712 is more favourable by ≈11 kJ mol^−1^, which is compensated only by a ≈8 kJ mol^−1^ more unfavourable Δ*H*^‡^_ass_. Considering that the dissociation free energy barrier, Δ*G*^‡^_diss_, is practically identical for the parent and Me-Ile2712 peptides (≈64 kJ mol^−1^), the slightly more favourable *T*Δ*S*^‡^_ass_ gives rise to the 3 kJ mol^−1^ decrease in Δ*G*_bind_ for Me-Ile2712 (Fig. 5c–e). Taken together, these data suggest that entropy-driven lowering of the association activation barrier arising from an increase in the free energy of the *N*-methylated peptide (relative to the parent SIM) represents a plausible explanation for the increased affinity for Me-Ile2712.

## Conclusions

We have performed a systematic backbone *N*-methylation scan of a 13-residue SIM peptide and assessed the effects on SUMO binding using a combination of competition fluorescence anisotropy and relaxation-based NMR experiments. At seven positions in the sequence, binding was abrogated or adversely affected, whereas at six positions binding was unaffected or improved. In instances where binding was diminished, this could be readily ascribed to the methylated peptide being unable to adopt a stable bound conformation. In instances where methylation did not affect, or, improved binding, this could be ascribed to faster binding to SUMO. Interestingly, this occurred for both hot-spot and flanking residues from the SIM peptide.

**Fig. 6:**
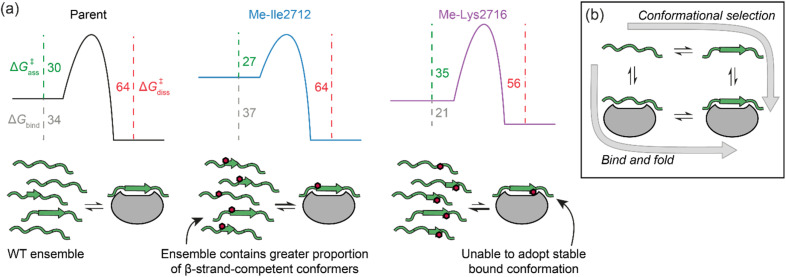
(a) Potential free energy diagrams together with schematics that could explain the data presented in Table 2. Grey, green and magenta dashed lines represent Δ*G*_bind_, Δ*G*^‡^_ass_, and Δ*G*^‡^_diss_, respectively, and their values are given in kJ mol^−1^ at 25 °C (note: we show the parent and Me-Ile2712 bound complexes as isoenergetic on the basis of their HSQC bound state spectra and Δ*G*^‡^_diss_); (b) schematic illustrates the extremes of conformational selection and bind-and-fold protein binding mechanisms.

In setting these results within the context of potential molecular mechanisms of recognition, we note the following additional considerations. First, there are likely subtle effects, such as a small increase in hydrogen-bond accepting ability of the carbonyl that might be expected upon *N*-methylation of the peptide bond. In turn, this would be anticipated to increase binding enthalpy. In addition, we cannot exclude the possibility that increased hydrophobicity, differential solvation of the methylated peptides, or subtle changes in bound conformation influence binding kinetics and affinity. Nonetheless, the entropy-driven increase in *k*_on_ rates that we observe lead us to conclude that increased affinities are caused by restricting the accessible conformational space of the *N*-methylated peptides. We simulated Ramachandran plots, which show that *N*-methylation significantly limits the phi(*ϕ*)/psi(*ψ*) angles accessible to residues on either side of the methylation site, such that residues are limited to extended or turn-like conformations (*i.e.*, excluded from α-helical space). However, NMR analyses suggest the unbound peptides do not adopt a defined conformation in the absence of SUMO. We contend that the overall ensemble of SIM conformers has a higher ground state energy and that this lowers the entropy of activation for SUMO binding (see free energy diagrams in Fig. 6a). We note that the polyproline-II conformation is somewhat intermediate between the α-helix and β-strand conformations,^46^ and that *N*-methylation is known to restrict the conformational space of peptide backbones.^29,30^ Thus, whilst the methylated peptides cannot be considered as pre-organized for SUMO binding, the ensemble is expected to disfavour α-space and thus favour β-space localized around the *N*-methylated residue so is primed or predisposed towards SUMO recognition. Previously, pre-organization of a peptide that recognises its target through a bind-and-fold^20^ mechanism (Fig. 6b) was shown not to enhance affinity for its target, because constraining limits “the number of ways to bind”,^47^ whilst for a peptide which recognises its target through conformational selection (Fig. 6b),^20^ constraining should increase affinity.^13^ Given *N*-methylation does not seem to induce a specific extended conformer and instead favours an ensemble of conformers that are compatible with binding, the effect observed here may represent a useful strategy to modulate thermodynamic and kinetic parameters of binding for ligands which bind their target through conformational selection or bind-and-fold mechanisms. Modulating peptide binding by tuning the entropy of activation/binding represents an untapped approach for design of peptidomimetic ligands. Whilst the overall effects on affinity observed here are small, methylation is known to confer proteolytic resistance and improved permeability, thus employing this modification within the toolkit for optimizing peptidomimetics may prove useful for development of therapeutically relevant PPI inhibitors. Establishing design rules that predict where an *N*-methyl group should be placed will require a larger data set; excluding amide NH's involved in hydrogen bonding or where a steric clash with the protein target would be envisioned to reduce the number of *N*-methyl variants that should be explored, however our results imply that *N*-Methylation may sterically influence local (Val2710) and remote (*via* Lys2716) side chain orientation to adversely impact target binding. Our future studies will explore backbone *N*-methylation to mechanistic analyses of other β-strand mediated PPIs and to explore the generality of our observations.

## Data availability

The data that support the findings of this study are available in the ESI† of this article.

## Author contributions

T. A. E., D. N. W., and A. J. W. conceived and designed the research program, E. E. C. and E. B. designed studies and performed the research, with support from T. K. K. The manuscript was written by E. E. C., T. K. K. and A. J. W. and edited into its final form by T. K. K., D. N. W., and A. J. W. with contributions from all authors.

## Conflicts of interest

There are no conflicts to declare.

## Supplementary Material

SC-015-D4SC02240H-s001
