# Supplementary material for: Understanding β-strand mediated protein–protein interactions: tuning binding behaviour of intrinsically disordered sequences by backbone modification
Source: Chem Sci. 2024 Jun 6;15(26):10237–45. doi: 10.1039/d4sc02240h (PMC11220606; doi:10.1039/d4sc02240h)
Supplement: SC-015-D4SC02240H-s001 [file SC-015-D4SC02240H-s001.pdf]

Electronic Supplementary Material (ESI) for Chemical Science.
This journal is © The Royal Society of Chemistry 2024

Understanding β-strand mediated protein-protein interactions: tuning binding behaviour of intrinsically disordered sequences by backbone modification

Emma E. Cawood, Emily Baker, Thomas A. Edwards, Derek N. Woolfson,* Theodoros K. Karamanos,* and Andrew J. Wilson,*

Dr Emma E. Cawood,† Prof Thomas A. Edwards,‡ Prof Andrew J. Wilson, Astbury Centre for Structural Molecular Biology, University of Leeds, Woodhouse Lane, Leeds LS2 9JT, UK

Dr Emma E. Cawood,† Prof Andrew J. Wilson, School of Chemistry, University of Leeds, Woodhouse Lane, Leeds LS2 9JT, UK

Dr Emily Baker, Prof Derek N. Woolfson, School of Biochemistry, University of Bristol, Medical Sciences Building, University Walk, Bristol BS8 1TD, UK, E-mail: d.n.woolfson@bristol.ac.uk

Dr Emily Baker, Prof Derek N. Woolfson, BrisSynBio, University of Bristol, Life Sciences Building, Tyndall Avenue, Bristol BS8 1TQ, UK

Dr Emily Baker, Prof Derek N. Woolfson, School of Chemistry, University of Bristol, Cantock's Close, Bristol BS8 1TS, UK

Prof Thomas A. Edwards, School of Molecular and Cellular Biology, University of Leeds, Woodhouse Lane, Leeds LS2 9JT, UK

Dr Theodoros Karamanos, Department of Life Sciences, Imperial College London, London, SW7 2BX, UK E-mail: t.karamanos@imperial.ac.uk

Prof Andrew J. Wilson, School of Chemistry, University of Birmingham, Edgbaston, Birmingham B15 2TT, UK, E-mail: a.j.wilson.1@bham.ac.uk

Prof Thomas A Edwards, College, of Biomedical Sciences, Larkin University, 18301 N Miami Ave #1, Miami, FL 33169

†Current address: The Francis Crick Institute, 1 Midland Rd, London NW1 1AT

Supporting Information

SI Figures and Tables ... 3
- Figure S1 ... 3
- Table S1. ... 4
- Figure S2 ... 5
- Figure S3 ... 6
- Figure S4 ... 6
- Figure S5 ... 7
- Figure S6 ... 8
- Figure S7 ... 9
- Figure S8 ... 10
- Figure S9 ... 11
- Figure S10 ... 12
- Figure S11 ... 12
- Figure S12 ... 13
- Figure S13 ... 14
- Figure S14 ... 15

Figure S15 16
Materials and methods 17
Peptide synthesis 17
HPLC purifications 17
Analytical HPLC 17
Mass spectrometry 17
Concentration determination 18
Fluorescence anisotropy 18
Protein expression 18
NMR spectroscopy – general information 18
^{15}N ZZ-exchange spectroscopy 19
^{15}N CPMG relaxation dispersion 19
Ramachandran Calculations 20
SIM peptide characterisation by analytical HPLC and mass spectrometry 21
References 25

SI Figures and Tables

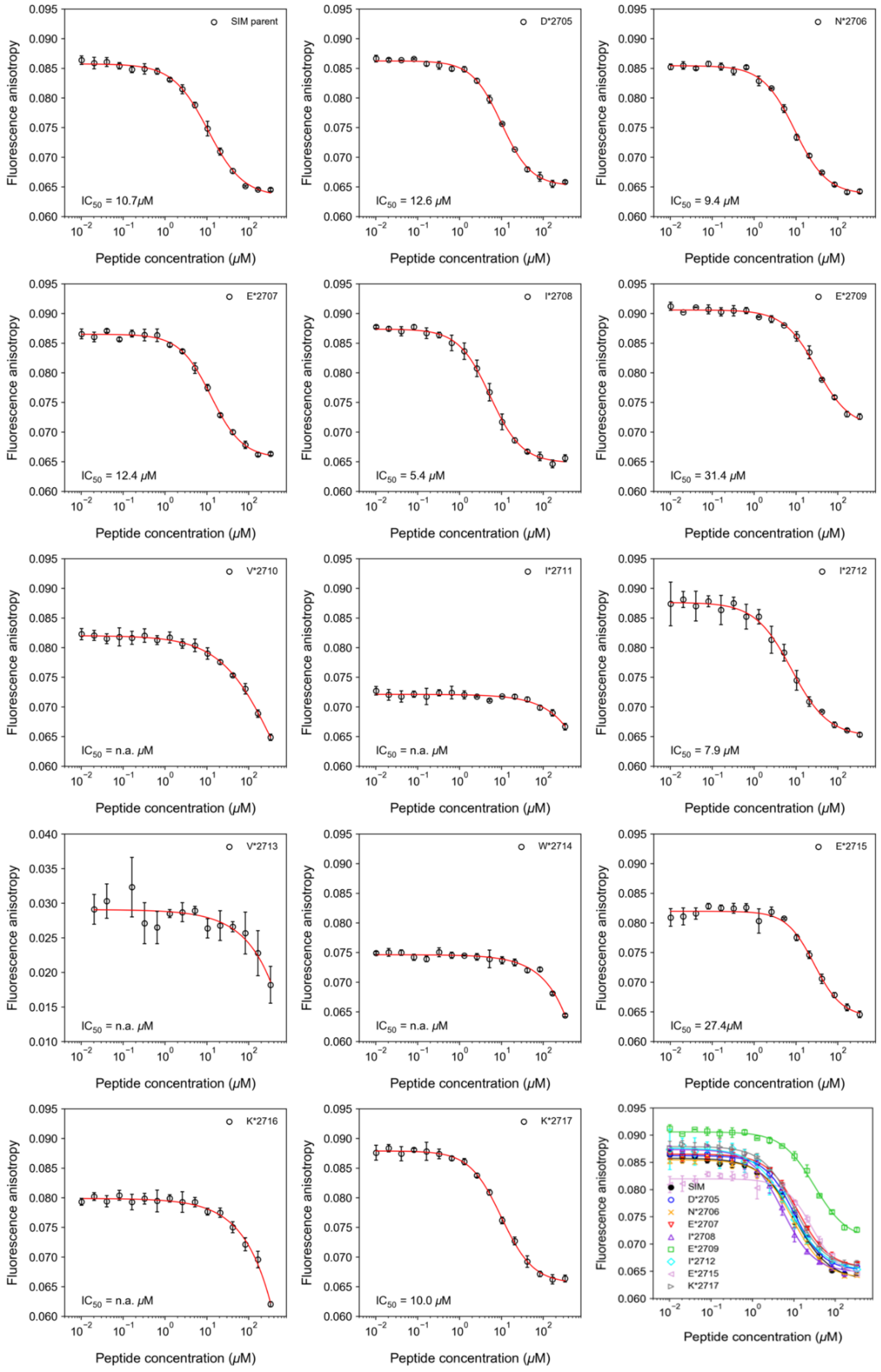

Figure S1. Fluorescence competition anisotropy data. The parent SIM peptide and its *N*-methylated variants were each titrated (333.3 μM to 10.2 nM) against a constant concentration of SUMO protein (3 μM) and FAM-SIM fluorescent tracer peptide (50 nM) in Tris-buffered saline (20 mM Tris, pH 8.0, 150 mM NaCl and 0.03% Triton-X). Note I: the different y axis scale for Me-Val2713 is a consequence of this data being acquired on a different instrument. Note II: these values are not directly comparable with those reported in our prior publication[1] where a different buffer composition was used: 20 mM Tris, 150 mM NaCl, 0.01 % Triton-X, pH 7.4 using 50 nM FAM PEG-SIM tracer and 3 μM SUMO protein.

Table S1. Inhibitory potencies for *N*-methylated SIM peptides to SUMO. IC_{50} values were determined through competition FA experiments (20 mm Tris, 150 mM NaCl, 0.03% Triton X, pH 8.0).

Peptide name	IC_{50} at 20 °C (μM)
Me-Asp2705	12.6 ± 0.5
Me-Asn2706	9.4 ± 0.5
Me-Glu2707	12.4 ± 0.8
Me-Ile2708	5.4 ± 0.3
Me-Glu2709	31.4 ± 3.0
Me-Val2710	> 100
Me-Ile2711	> 300
Me-Ile2712	7.9 ± 1.0
Me-Val2713	> 100
Me-Trp2714	> 100
Me-Glu2715	27.4 ± 1.9
Me-Lys2716	> 100
Me-Lys2717	10.0 ± 0.4
Parent SIM	10.7 ± 0.7

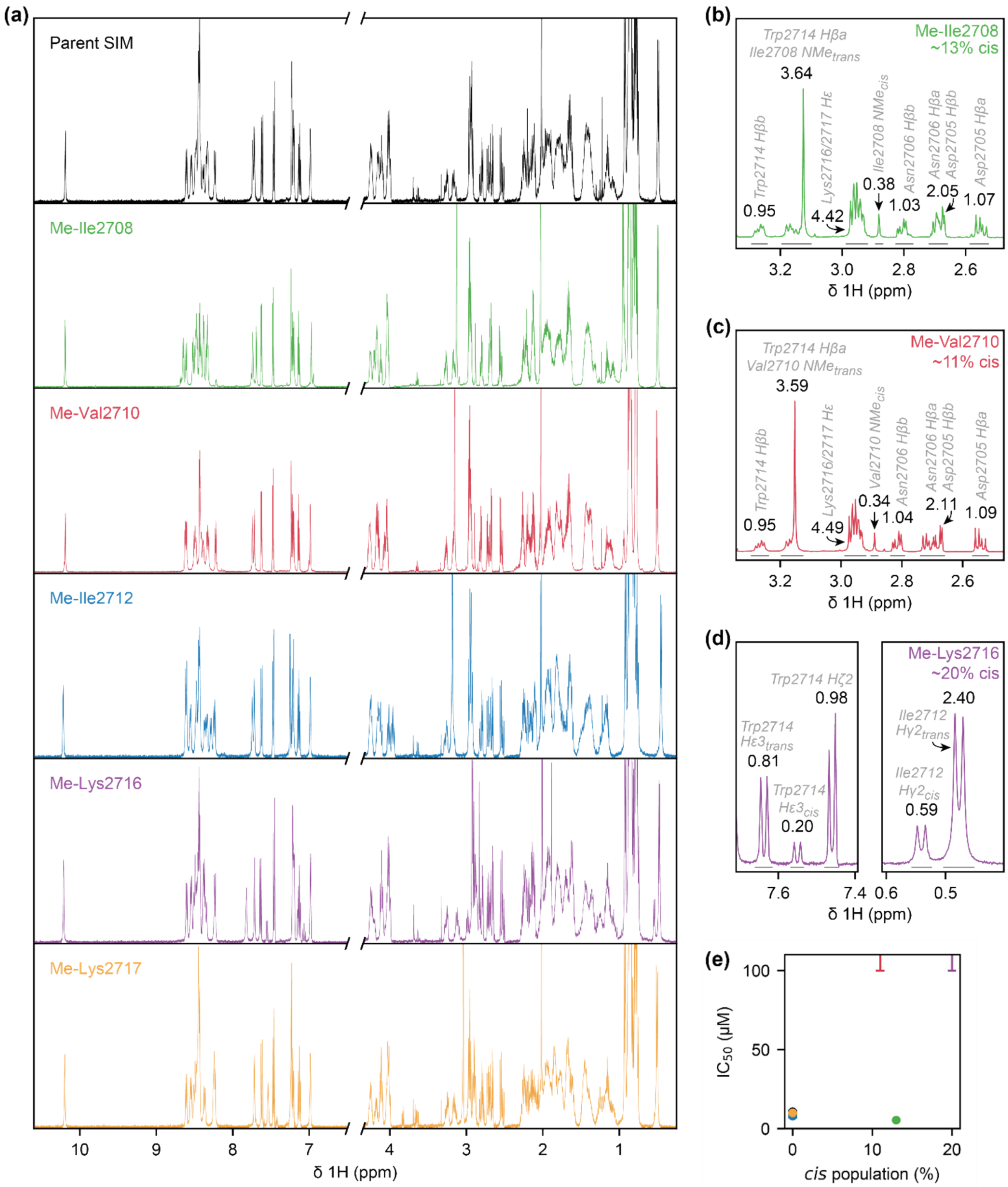

Figure S2. ^{1}H spectra acquired for the parent SIM peptide and *N*-methylated variants of interest show that while some variants have detectable *cis* populations (assigned based on: (i) comparison to parent SIM shifts, and (ii) NOEs) around the *N*-methylated peptide bond, this does not account for the observed changes in SUMO binding affinity. (a) ^{1}H spectra were acquired at 5 °C with 500 μM peptide in 20 mM sodium phosphate pH 7.4, 0.02% NaN_3, at 750 MHz (Me-Ile2708, Me-Val2710) or 500 MHz (all other peptides). (b)-(d) ^{1}H assignments (see Table S2) that were obtained through various ^{1}H-^{1}H correlation experiments (TOCSY, COSY, and NOESY) indicated that Me-Ile2708, Me-Val2710, and Me-Lys2716 had a detectable population of the *cis* isomer at the methylated amide bond. Integration of the relevant signals in the ^{1}H spectra allowed the approximate *cis* population to be calculated. Integral regions are indicated by the grey lines under the peaks, and the relative areas of these regions are indicated. (e) While some of the *N*-methylated peptides exhibited a detectable population of the *cis* isomer (p_{cis}) (i.e., Me-Ile2708, Me-Val2710 (red) and Me-Lys2716), no correlation between p_{cis} and potency was observed.[2] The exact IC_{50} values for Me-Val2710 (red) and Me-Lys2716 (purple) could not be accurately determined in competition fluorescence anisotropy experiments (Fig. S1) but are known to be >100 μM.

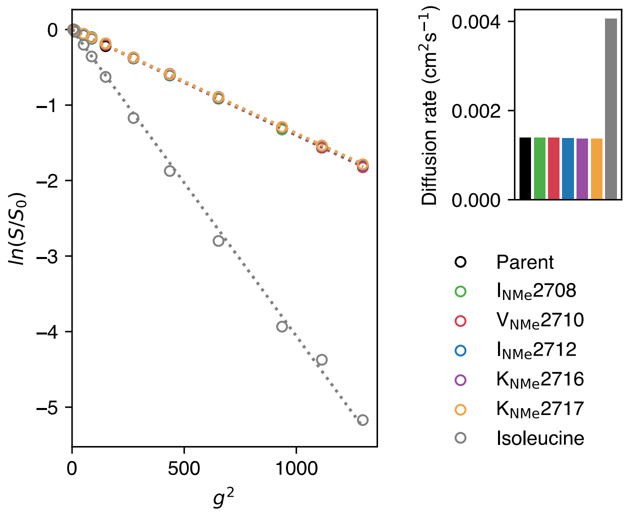

Figure S3. Pulsed field gradient diffusion NMR analysis on the parent SIM peptide and select *N*-methylated variants. Integral region = 0.7-1.0 ppm. None of the *N*-methylated variants exhibited any changes or difference in diffusion rate relative to the parent SIM peptide.

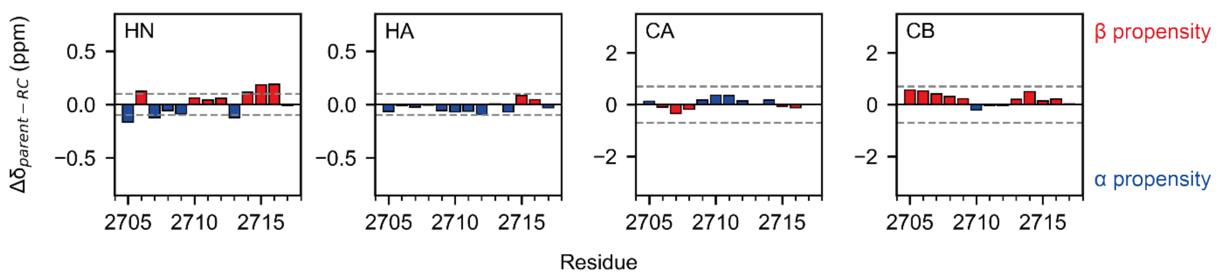

Figure S4. ^{1}H and ^{13}C Secondary chemical shifts of parent SIM peptide.

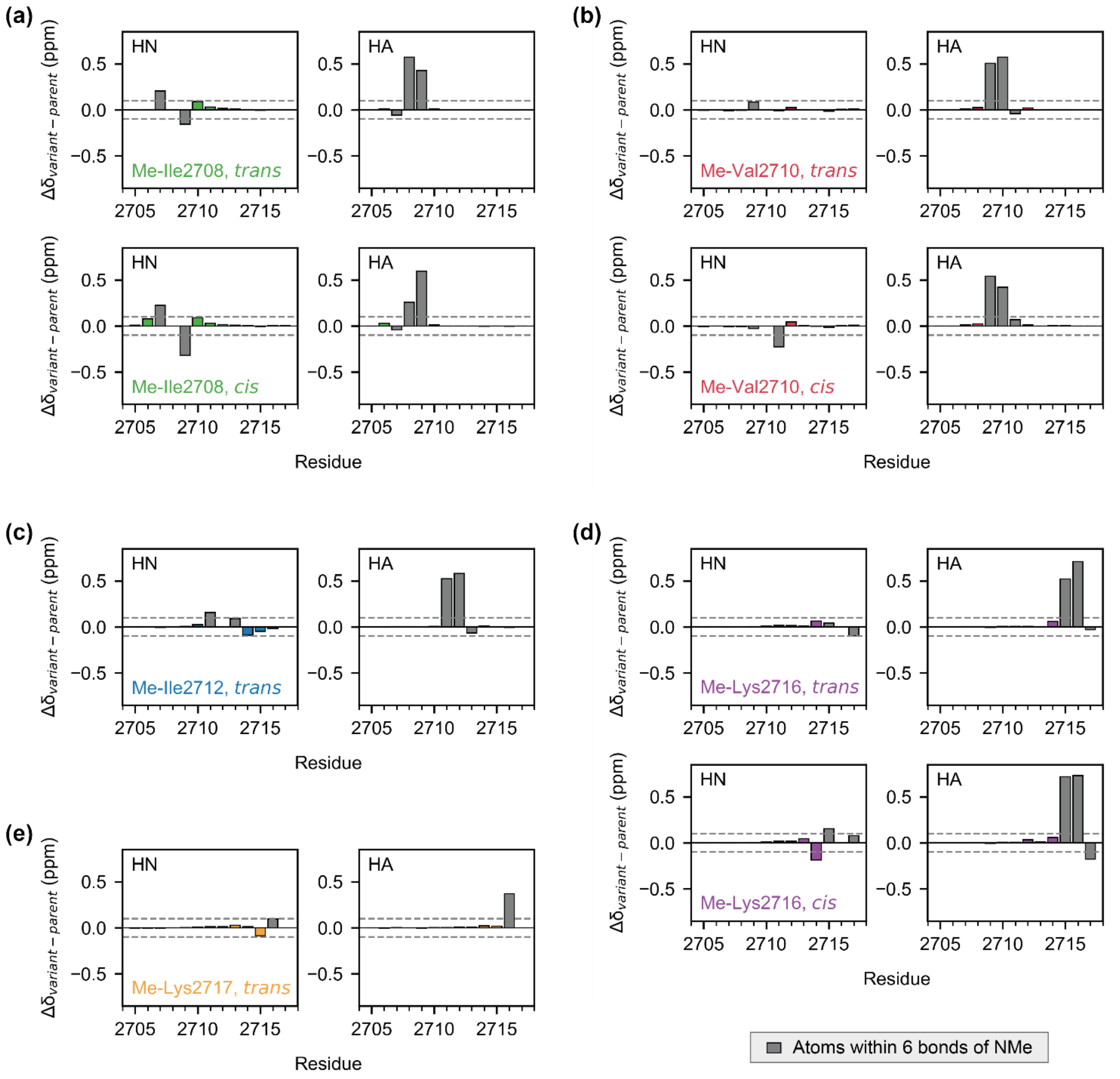

Figure S5. [1]H secondary chemical shifts differences between parent SIM and variant SIM peptides. Bars in grey indicate atoms within six bonds of the carbon of the introduced methyl group. The remaining bars are coloured according to the associated variant.

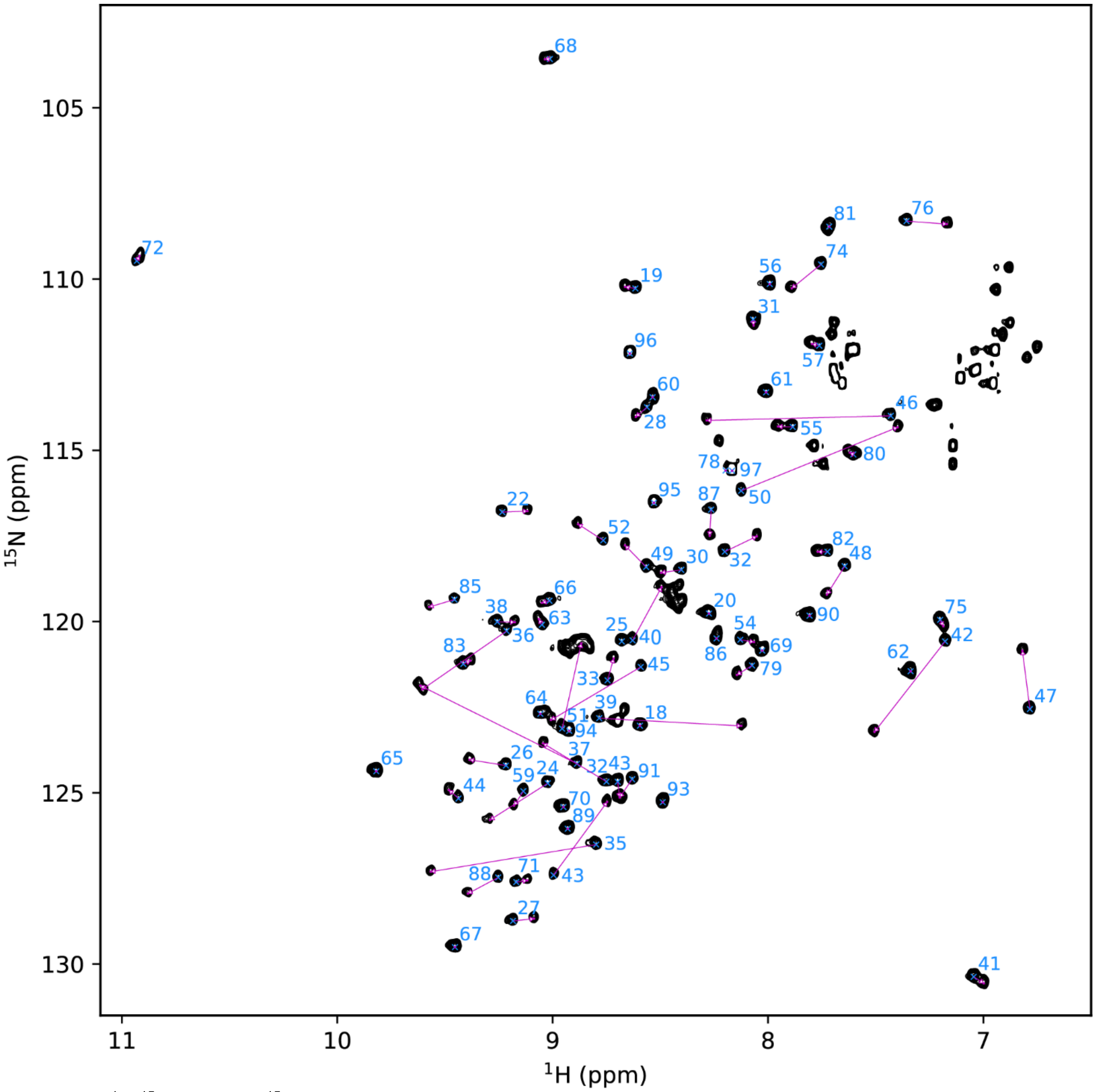

Figure S6. ^{1}H-^{15}N HSQC of ^{15}N-SUMO (250 μM) in the presence of the parent SIM peptide (125 μM), in 20 mM sodium phosphate pH 7.4, 150 mM NaCl, 5% D_2O, 2 mM DTT, 0.02% NaN_3, at 5°C, 750 MHz. Amide resonances are labelled by residue number, with two peaks being observed for certain residues due to slow exchange between the apo and SIM-bound states of SUMO. For these residues, the apo and bound peaks are linked by a pink line.

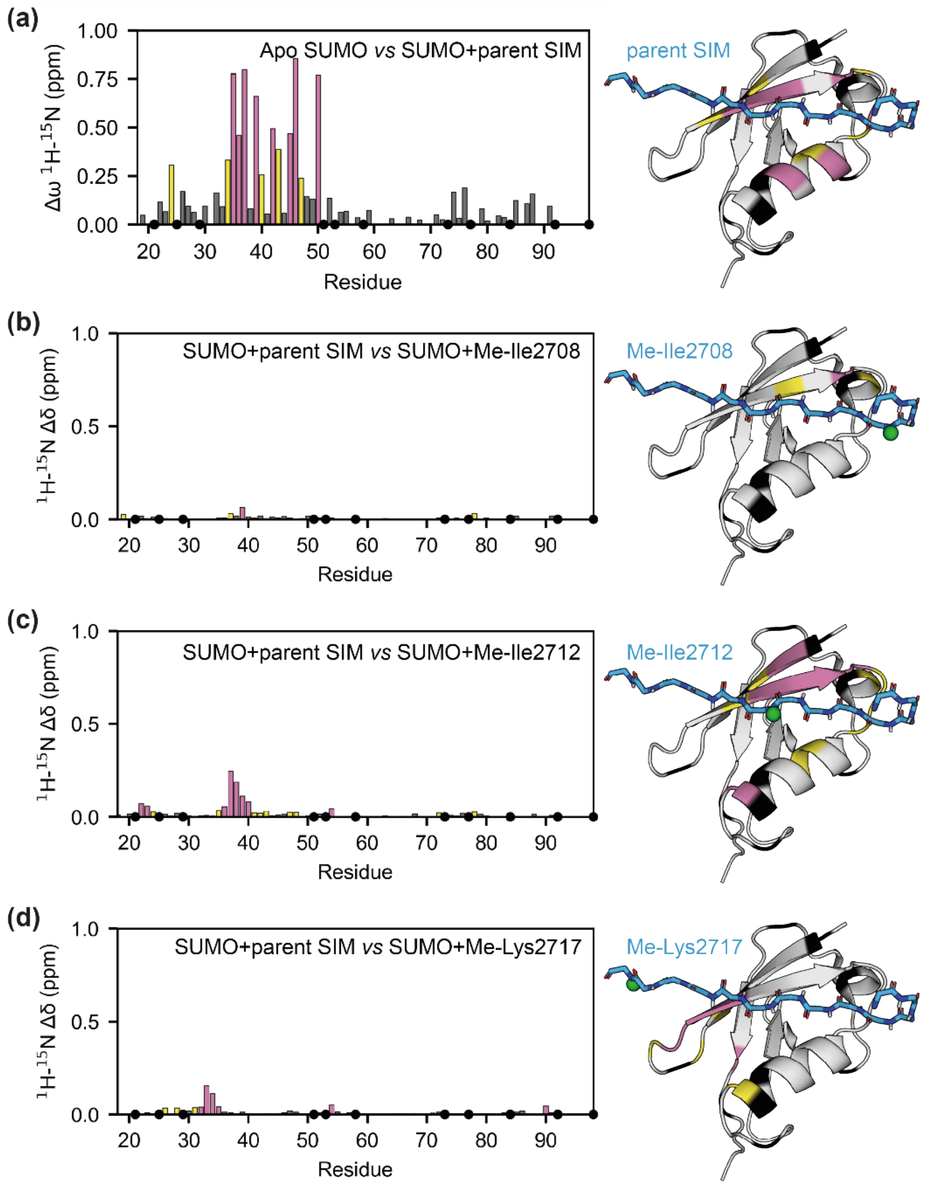

Figure S7. Chemical shift perturbation analysis for SUMO in the presence/absence of different SIM variants. In all cases combined ^{1}H-^{15}N chemical shift differences are shown. (a) Difference between unbound SUMO and SUMO bound to the parent SIM peptide. (b-d) Difference between SUMO bound to the parent SIM peptide and SUMO bound to either Me-Ile2708 (b), Me-Ile2712 (c), or Me-Lys2717 (d). In all cases, SUMO residues which could not be confidently assigned in both states are shown in black, while the remaining residues are coloured according to the magnitude of Δω, relative to relative to the standard deviation (σ) of the dataset (< 1σ, grey; 1-2σ, yellow; ≥2σ, pink). Mapping the magnitude of Δω onto to structure of SUMO (PDB 2LAS)[3] confirms that parent SIM (cyan) binds to SUMO in the canonical binding groove (between the α1 helix and β2 strand) (a), and that the three *N*-methylated SIM variants shown in this figure are likely to adopt the same canonical binding pose given that the only differences between the bound state chemical shifts of the various SIM/SUMO complexes occur at SUMO residues which lie adjacent to the *N*-methylated SIM residue (green in the structures) in the canonical bound state (b-d).

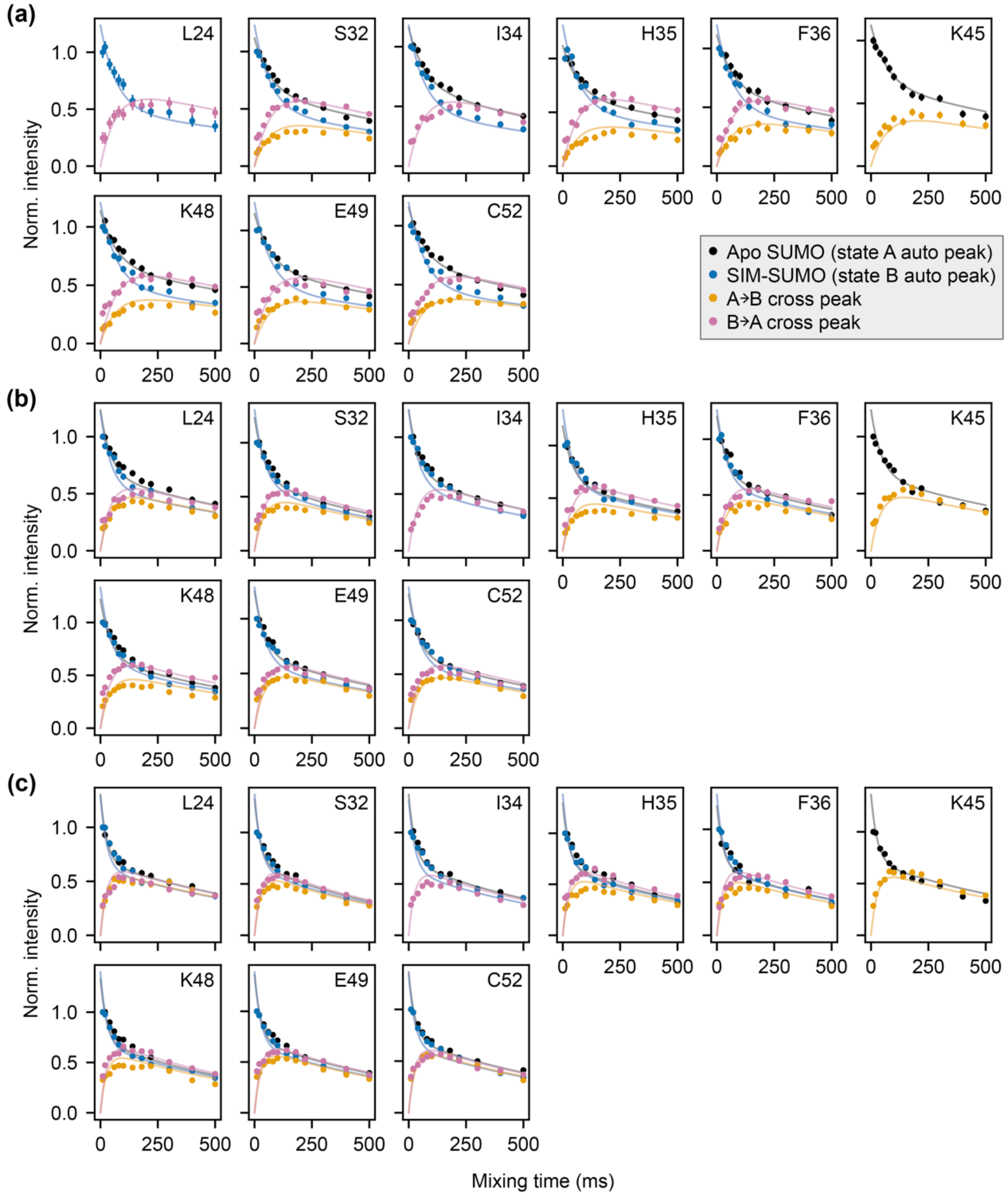

Figure S8. ^{15}N ZZ-exchange profiles as a function of mixing time for ^{15}N-SUMO (250 μM) in the presence of the parent SIM peptide (125 μM) at 5°C (a), 10°C (b), or 15°C (c). Filled circles represent the experimental data and error bars were calculated from the signal-to-noise ratio. Solid lines represent fits to a 2-state model (see Figure 3a).

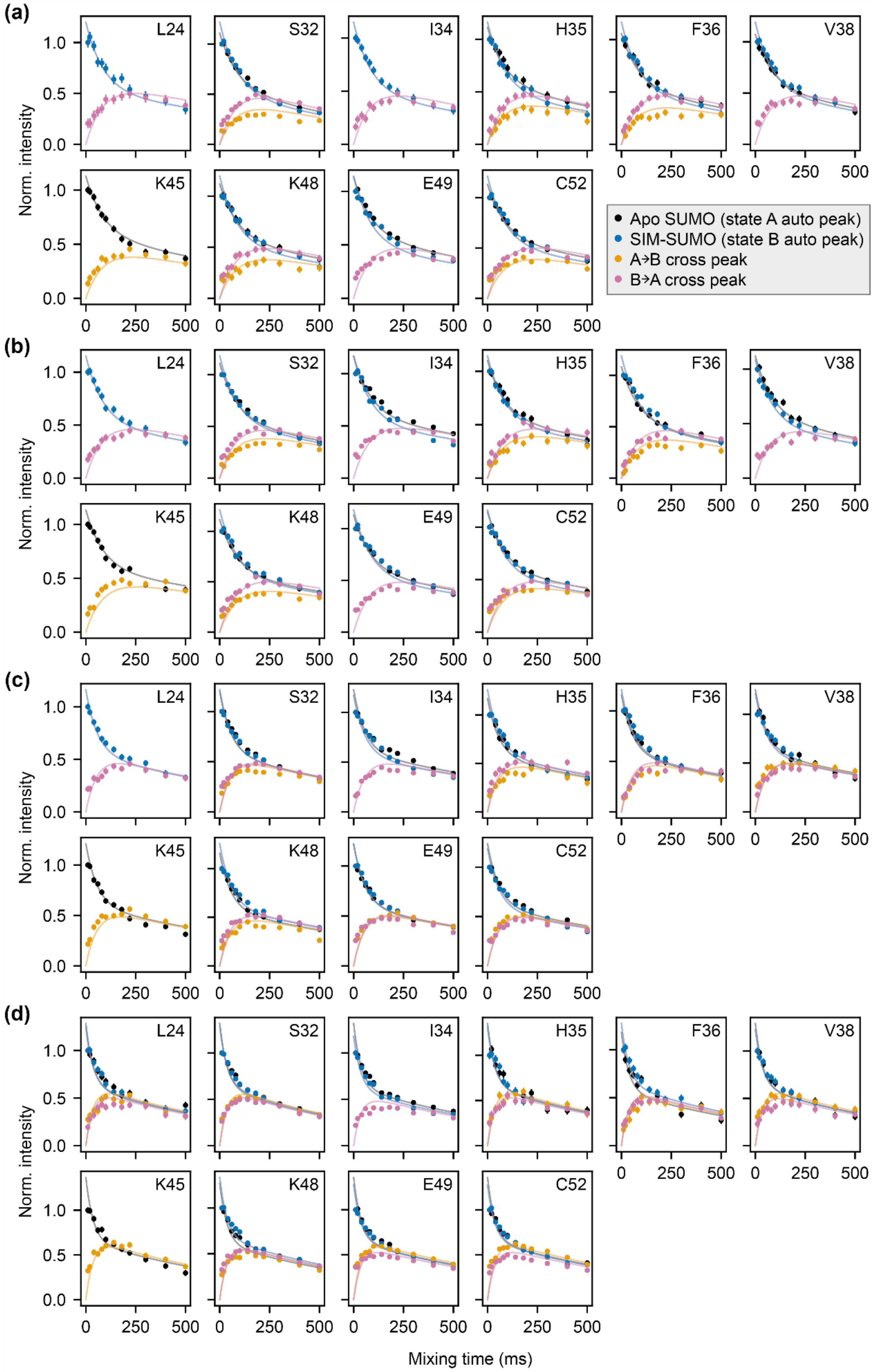

Figure S9. ^{15}N ZZ-exchange profiles as a function of mixing time for ^{15}N-SUMO (250 µM (a) or 240 µM (b-d)) in the presence of Me-Ile2712 SIM (125 µM) at 5°C (a, b), 10°C (c), or 15°C (d). Filled circles represent the experimental data and error bars were calculated from the signal-to-noise ratio. Solid lines represent fits to a 2-state model (see Figure 3a).

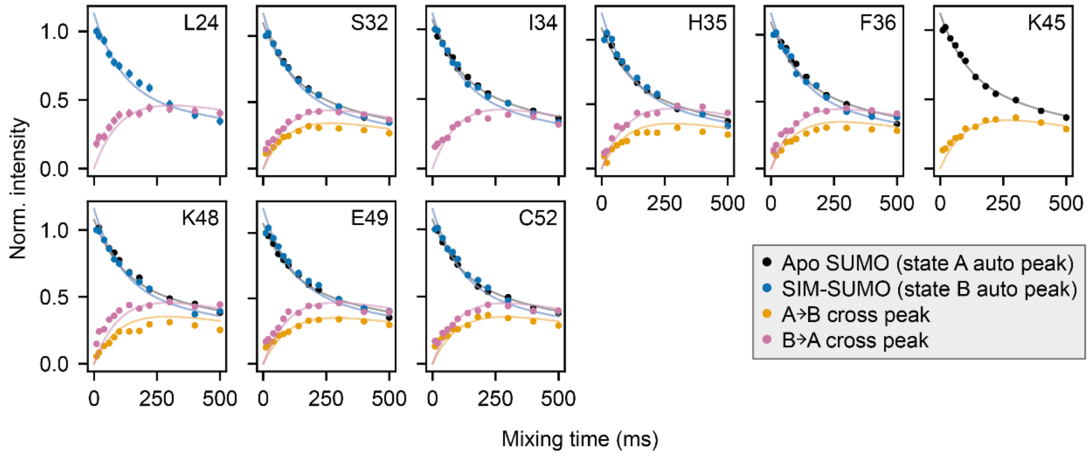

Figure S10. [15]N ZZ-exchange profiles as a function of mixing time for ^{15}N-SUMO (250 μM) in the presence of Me-Ile2708 SIM (125 μM) at 5°C. Filled circles represent the experimental data and error bars were calculated from the signal-to-noise ratio. Solid lines represent fits to a 2-state model (see Figure 3a).

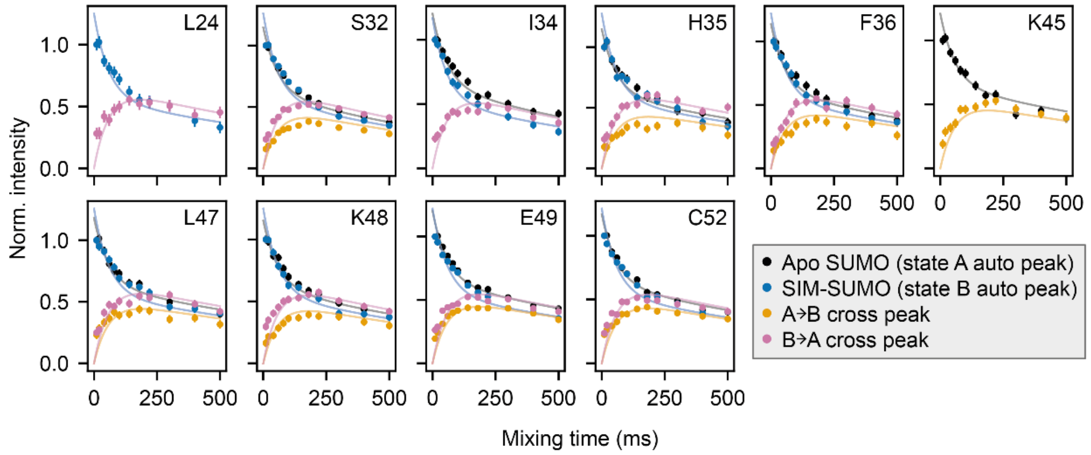

Figure S11. [15]N ZZ-exchange profiles as a function of mixing time for ^{15}N-SUMO (250 μM) in the presence of Me-Lys2717 SIM (125 μM) at 5°C. Filled circles represent the experimental data and error bars were calculated from the signal-to-noise ratio. Solid lines represent fits to a 2-state model (see Figure 3a).

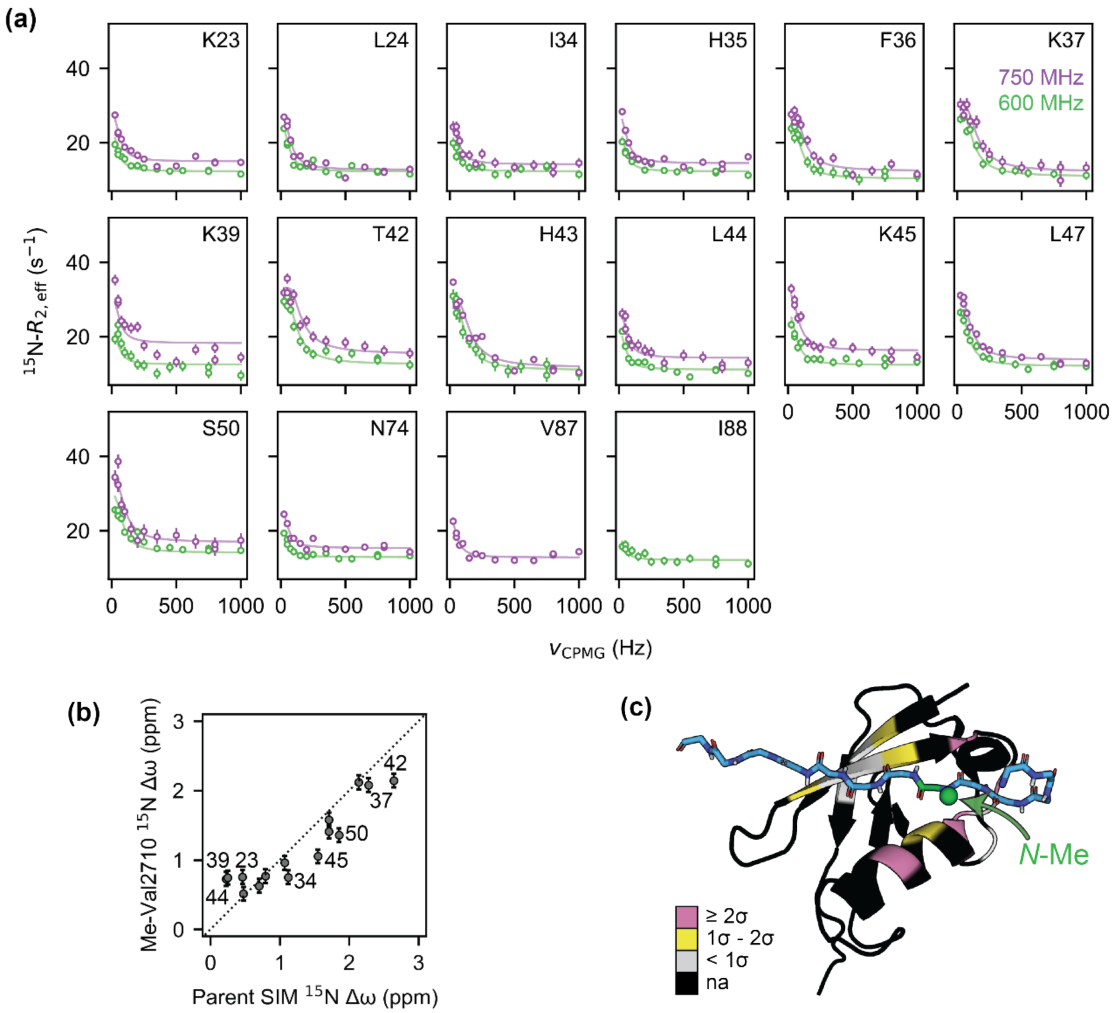

Figure S12. (a) ^{15}N-CPMG profiles for ^{15}N-SUMO (250 μM) in the presence of Me-Val2710 SIM (50 μM) at 5°C and 600 (green) or 750 (magenta) MHz. Circles represent the experimental data and solid lines represent fits to a 2-state model (see Figure 3a). (b) Correlation between the fitted chemical shifts of SUMO in the presence of Me-Val2710 and those observed directly from the ^{1}H-^{15}N HSQC spectrum of SUMO in the presence of parent SIM (Pearson correlation coefficient: 0.93). Residue numbers are shown above select datapoints. (c) Structure of the complex between SUMO and parent SIM (PDB: 2LAS), coloured by the chemical shift difference between the fitted chemical shifts of SUMO in the presence of Me-Val2710 and those observed directly for SUMO in the presence of parent SIM. The colour scheme is relative to the standard deviation (σ) of the dataset (<1σ, grey; 1-2σ, yellow; ≥2σ, pink). The site of N-methylation is shown as a green sphere.

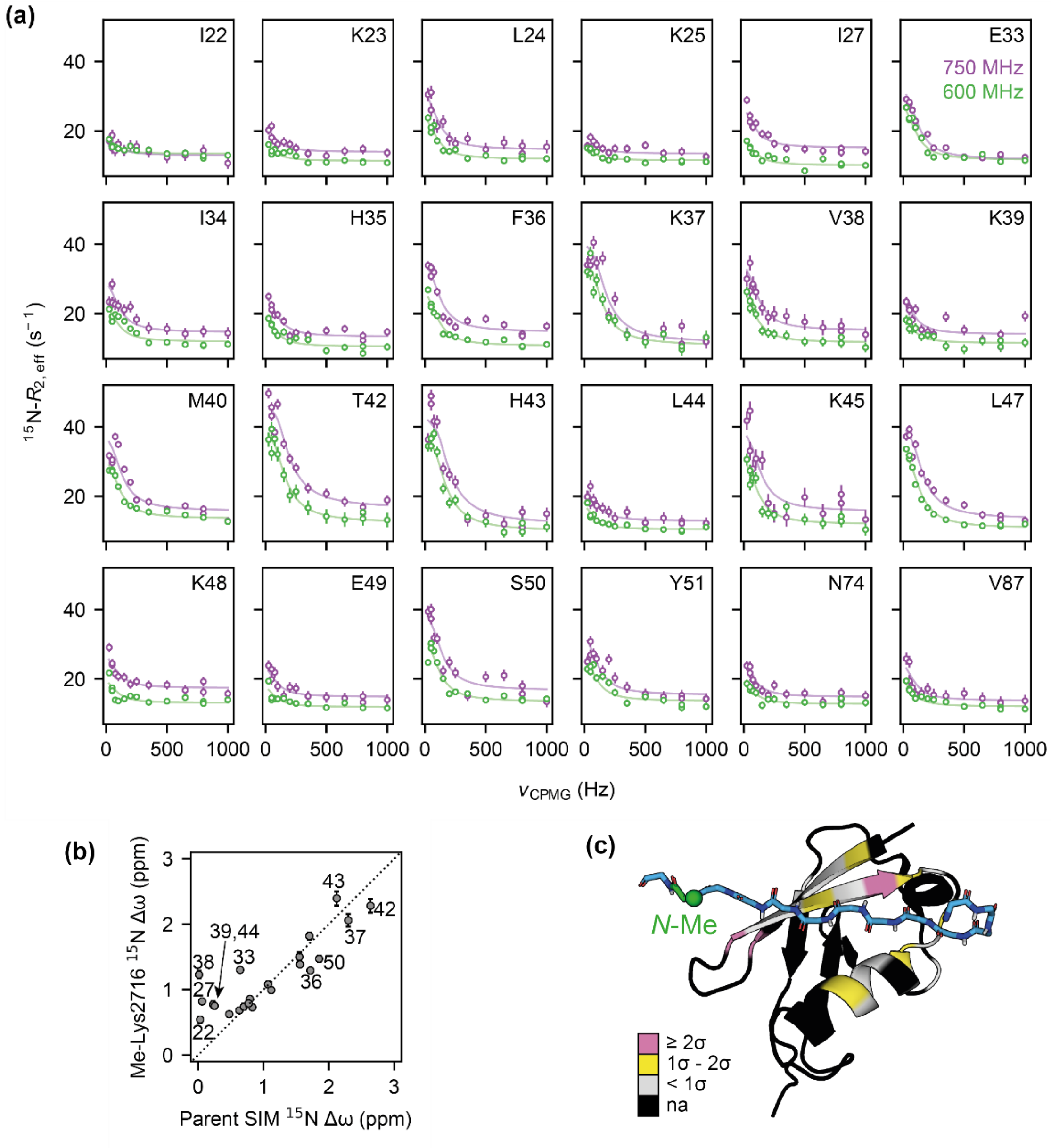

Figure S13. (a) ^{15}N CPMG profiles for ^{15}N-SUMO (250 μM) in the presence of Me-Lys2716 SIM (50 μM) at 5°C and 600 (green) or 750 (magenta) MHz. Circles represent the experimental data and solid lines represent fits to a 2-state model (see Figure 3a). (b) Correlation between the fitted chemical shifts of SUMO in the presence of Me-Lys2716 and those observed directly from the ^{1}H-^{15}N HSQC spectrum of SUMO in the presence of parent SIM (Pearson correlation coefficient: 0.86). Residue numbers are shown above select datapoints. (c) Structure of the complex between SUMO and parent SIM (PDB: 2LAS), coloured by the chemical shift difference between the fitted chemical shifts of SUMO in the presence of Me-Lys2716 and those observed directly for SUMO in the presence of parent SIM. The colour scheme is relative to the standard deviation (σ) of the dataset (<1σ, grey; 1-2σ, yellow; ≥2σ, pink). The site of N-methylation is shown as a green sphere.

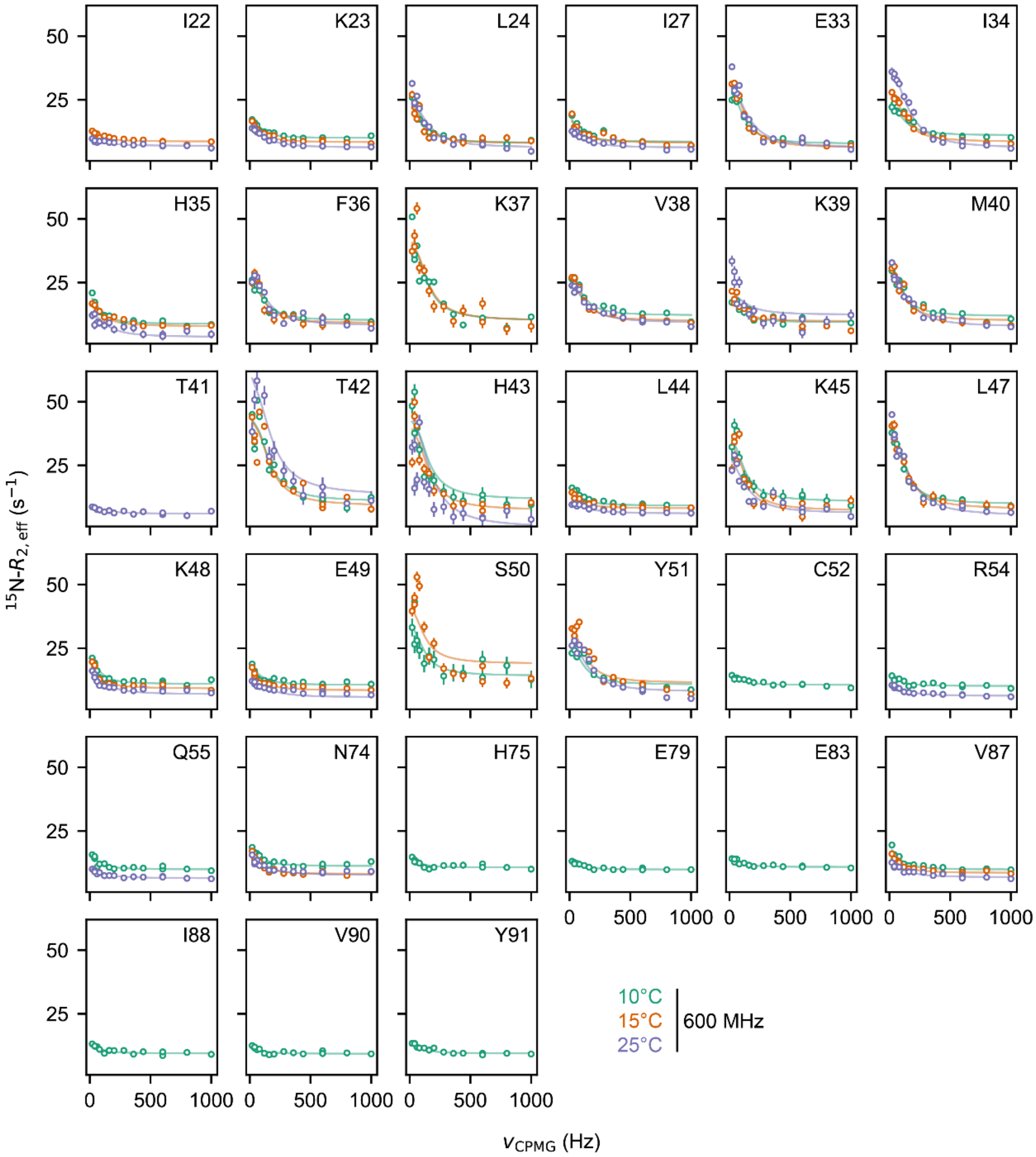

Figure S14. ^{15}N CPMG profiles of SUMO (250 µM) in the presence of Me-Lys2716 (50 µM) acquired at 600 MHz and 10 °C (green), 15 °C (red) or 25 °C (blue).

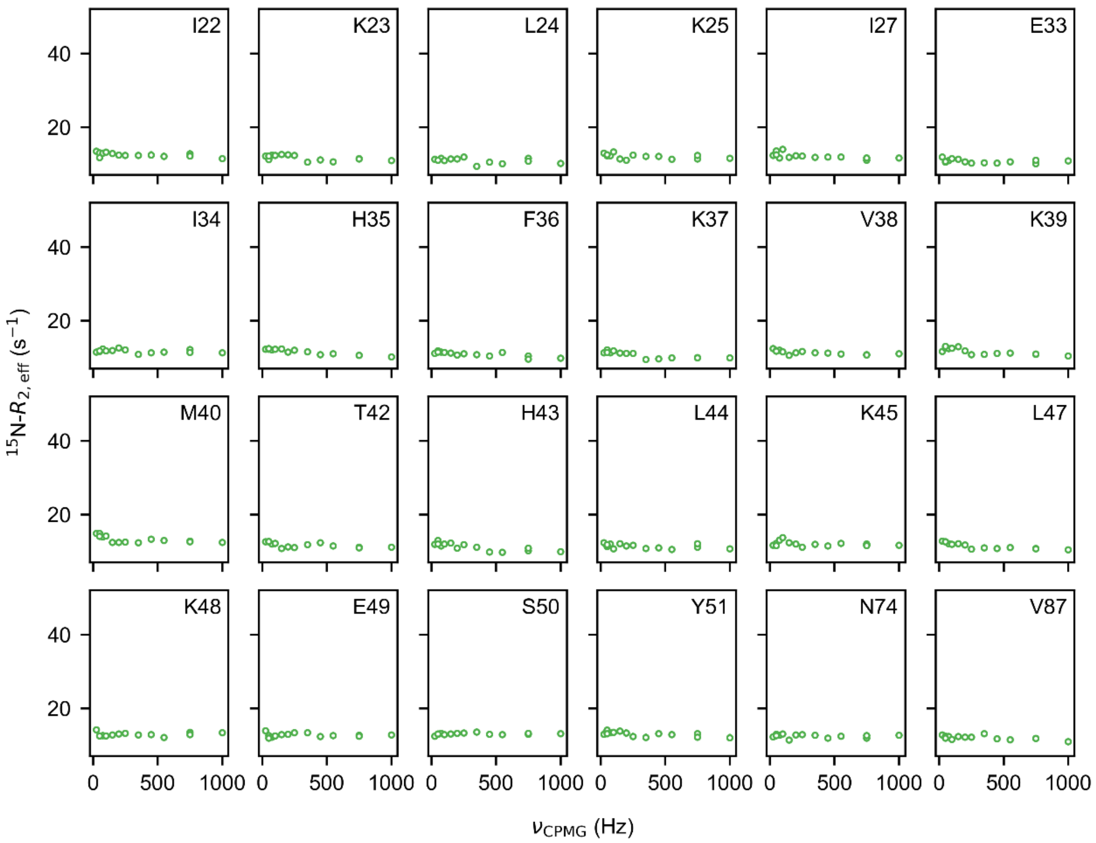

Figure S15. In the absence of a SIM peptide, SUMO (250 μM) shows no CPMG curves, indicating that the CPMG profiles observed for SUMO in the presence of Me-Val2710 (Fig. S11) and Me-Lys2716 (Fig. S12) report on peptide binding. Data was acquired at 600 MHz, 5 °C.

Materials and methods

Peptide synthesis

Peptides were synthesised using a Liberty Blue microwave-assisted automated peptide synthesiser (CEM Corporation) on a rink amide 100-200 mesh MBHA resin (Merck) using standard Fmoc-coupling chemistry. Double Fmoc deprotection steps were performed with 5:20:75 vol% formic acid (Fisher Scientific):morpholine (Sigma Aldrich):DMF (Cambridge Reagents) for 30 sec at 75°C (125 W) then 1 min at 90°C (30 W) for each deprotection; 5% formic acid prevented aspartimde formation. Double peptide couplings followed, using 1 M *N*,*N*′-diisopropylcarbodiimide (Sigma Aldrich) as the activator and 0.5 M Oxyma (Biosynth Carbosynth) as the activator base, for 30 sec at 75°C (125 W) then 4 min at 90°C (30 W) for each coupling, except in the case of the *N*-methylated and their succeeding amino acids (as synthesised *C*→*N*), for which extended coupling times of 10 mins at 90°C (30 W) were used. Peptides were acetylated at their *N*-termini via 0.5 mL pyridine (Fisher Scientific) with 0.25 mL acetic anhydride (BDH Laboratories) for 15 mins under agitation. For Me-Asp2705, the acetylation step was performed 3 times, with thorough washing of the resin between. Peptides were cleaved from the resin in 2.5:97.5 vol% triisopropylsilane (Acros Organics):trifluoroacetic acid (Sigma Aldrich) for 90-120 min under agitation. It was necessary to omit water during peptide cleavage as this sometimes resulted in peptide degradation. The cleaved peptide mixture was then filtered from the resin and the volume reduced to <5 mL using a flow of nitrogen. Peptides were obtained by precipitation from the addition of diethylether (VWR Chemicals) followed by centrifugation to remove the supernatant. The peptide pellet was then dissolved in 1:1 vol% H_2O:acetonitrile (Fisher Scientific) and lyophilised to yield the crude peptides as white powders.

HPLC purifications

Peptides were purified using linear gradients of buffer B vs. buffer A on a JASCO HPLC system. Me-Lys2716, Me-Lys2717 were purified using basic buffers (A = 25 mM ammonium bicarbonate in H_2O, B = 100% acetonitrile). All other peptides were purified using acidic buffers (A = 0.1 vol% TFA in H_2O, B = 0.1 vol% TFA in acetonitrile) and a 50°C column oven. To obtain sufficient resolution, reverse-phase columns ranging in size from a preparatory C18 Vydac column (250 x 22 mm, particle size = 10 μM, pore size = 300 Å, Grace), through a semi-preparatory C18(2) Luna column (150 x 10 mm, particle size = 5 μM, pore size = 100 Å, Phenomenex) or a C8(2) Luna column (250 x 10 mm, particle size = 5 μM, pore size = 100 Å, Phenomenex), to an analytical C18 Kinetex column (100 x 4.6 mm, particle size = 5 μM, pore size = 100 Å, Phenomenex) were employed as required. Generally, ≤1 mg of crude peptide was dissolved (either in 20:80 vol% buffer B:A or 20:60:20 vol% buffer B:A:DMSO) and purified at a time, regardless of column size. A preparatory flow cell was used in all cases. Chromatograms were recorded at 220 nm and 280 nm simultaneously.

Analytical HPLC

Peptide purity was confirmed using a JASCO HPLC system equipped with a reverse-phase analytical C18 Kinetex column (100 x 4.6 mm, particle size = 5 μM, pore size = 100 Å, Phenomenex) and an analytical flow cell, and using gradients of acidic buffer B (0.1 vol% TFA in acetonitrile) vs. buffer A (0.1% TFA in H_2O). Chromatograms were recorded at 220 nm and 280 nm simultaneously.

Mass spectrometry

Peptide identities were confirmed using either a Waters Synapt G2S IMS Q-TOF mass spectrometer, in positive ion mode, equipped with an Advion Nanomate Triverser nanospray source (peptides were dissolved in 1:1 vol% H_2O:acetonitrile and infused at 1.4 kV), or an ultrafleXtreme II MALDI-TOF instrument (Bruker) in reflector positive or negative ion mode from peptides co-crystalised with dihydroxybenzoic acid matrix (Sigma Aldrich) on a ground steel plate.

Concentration determination

Concentrations were determined using a NanoDrop 2000 spectrophotometer (Thermo Scientific) using the known extinction coefficient of 5'6-fluoroscein ($\varepsilon_{494\ nm}$ = 68,000 M^{-1} cm^{-1}) for FAM-SIM, or tryptophan for all other peptides ($\varepsilon_{280\ nm}$ = 5690 M^{-1} cm^{-1}). The concentration of SUMO was determined using the calculated extinction coefficient of $\varepsilon_{280\ nm}$ = 4470 M^{-1} cm^{-1} based on the protein sequence.

Fluorescence anisotropy

Enough peptide was purified to enable titration of stock solutions (in H_2O) to ~pH 8.0 before the appropriate amount (determined by peptide concentration) was then aliquoted and freeze-dried to allow reconstitution at 2 mM in 500 µL peptide buffer (20 mM Trizma® base (Sigma Aldrich), 150 mM NaCl (Fisher Scientific), pH 8.0). The pH and concentration were then confirmed.

Competition binding assays were performed in triplicate within 384-well plates (Greiner Bio-one). Competitor peptides serially diluted and then SUMO protein and FAM-SIM tracer added to sample wells (333.3 µM > 10.2 nM, 3 µM, 50 nM final concentrations of competitor, SUMO and tracer respectively). Control wells lacked tracer. The plates were kept in the dark for at least 30 min before fluorescence polarisation was measured. For all peptides other than Me-Val2713, this was done using a Perkin Elmer CLARIOstar plate reader (BMG Labtech), with excitation at 482 nm (bandwidth = 16 nM) and emission at 530 nm (bandwidth = 40 nm). For Me-Val2713, the data were collected using a Spark 10M plate reader (Tecan), with excitation at 485 nm (bandwidth = 20 nM) and emission at 535 nm (bandwidth = 25 nm).

Perependicular (P) and parallel (S) intensities of the control wells were averaged and deducted from each corresponding sample to give the corrected values, P_{corr} (where $P_{corr} = P_{sample} - P_{av.\ control}$) and S_{corr} (where $S_{corr} = S_{sample} - S_{av.\ control}$). The total sample intensities (I) and anisotropies (r) were then calculated as follows:

$$I = (2 \times P_{corr}) + S_{corr}$$

$$r = (S_{corr} - P_{corr}) / I$$

Fluorescence anisotropy values were plotted against competitor peptide concentrations and fit to a logistic function of the form $Y = A2 + (A1 - A2) / (1 + (x/x_0)^p)$ for determination of half-maximal inhibitory concentrations (IC_{50}) of the peptide competitors (A2 is the upper asymptote and A1 the lower asymptote).

Note: To ensure a set of self-consistent IC_{50} values for comparison, experiments were performed using a single batch of protein given subtle batch dependent variations in active concentration of protein. The values obtained here are not directly comparable with those reported in our prior publication[1] where a different buffer composition was used (20 mM Tris, 150 mM NaCl, 0.01 % Triton-X, pH 7.4 using 50 nM FAM PEG-SIM tracer and 3 µM SUMO protein).

Protein expression

^{14}N-labelled SUMO (human GlyThrHisMet-$SUMO_{18\text{-}97}$) was expressed and purified as previously described from a pet19b His-TEV-$SUMO1_{18\text{-}97}$ construct.[1] ^{15}N-labelled SUMO was expressed and purified following the same protocol as above, with the expression being carried out in M9 minimal media supplemented with ^{15}N-NH_4Cl.

NMR spectroscopy – general information

All NMR spectra acquired for isolated peptide samples were natural abundance spectra acquired at 5 °C, with 500 µM peptide in 20 mM sodium phosphate pH 7.4, 0.02% NaN_3, on 750 or 500 MHz Bruker NMR spectrometers. Random coil reference shifts for the parent SIM sequence (DNEIEVIIVWEKK) were obtained using the Poulsen IDP/IUP Random Coil Chemical Shifts (can be accessed at https://spin.niddk.nih.gov/bax/). These random coil shifts are calculated using neighbour correction factors,[4] and pH- and temperature-corrections.[5]

All NMR spectra acquired using ^{15}N-labelled protein samples were acquired at 5 °C, in 20 mM sodium phosphate pH 7.4, 150 mM NaCl, 0.02% NaN_3, on 750 or 600 MHz Bruker NMR spectrometers.

Protein and peptide NMR spectra were processed with NMRPipe and analysed in CcpNmr analysis.

Pulsed field gradient NMR

Diffusion NMR measurements on SIM peptides alone were measured in an interleaved manner on a 750 MHz Bruker spectrometer using pulse sequences that employ bipolar gradients. Data were recorded at 5 °C, with a 400 ms diffusion delay.

^{15}N ZZ-exchange spectroscopy

^{15}N ZZ-exchange experiments were performed in an interleaved manner as described previously[4] using mixing times between 10 and 500 ms. Data were fit to a simple 2-state model:

$$A \underset{k_{off}}{\overset{k_{on}^{app}}{\rightleftarrows}} B \qquad \text{(Scheme 1)}$$

where state A represents unbound SUMO and state B represents SUMO bound to SIM. The time-evolution of the longitudinal magnetisations during mixing are described by the corresponding McConnel equations:[4]

$$\frac{dM_z^{aa}}{dt} = k_{off}M_z^{ab} - M_z^{aa}(k_{on}^{app} + R_1^a)$$

$$\frac{dM_z^{bb}}{dt} = k_{on}^{app}M_z^{ba} - M_z^{bb}(k_{off} + R_1^b)$$

$$\frac{dM_z^{ab}}{dt} = k_{on}^{app}M_z^{aa} - M_z^{ab}(k_{off} + R_1^b)$$

$$\frac{dM_z^{ab}}{dt} = k_{off}M_z^{bb} - M_z^{ba}(k_{on}^{app} + R_1^a)$$

where M_z^{aa} is the *z*-magnetization of unbound SUMO, M_z^{bb} is the *z*-magnetization of bound SUMO, and M_z^{ab}, M_z^{ba} are the *z*-magnetizations of the corresponding exchange cross-peaks. R_1^a, R_1^b are the longitudinal relaxation rates for states A and B, and k_{on}^{app}, k_{off} are the pseudo-first order association and dissociation rates, respectively. Simple analytical solutions to the above equations are available[4] and were used to fit the ZZ-exchange data. Fitting parameters included k_{on}^{app}, k_{off}, residue specific R_1^a, R_1^b rates, and scaling factors I_{aa}, I_{bb} that incorporate any differences in the relaxation rates of the bound and unbound species. Scaling factors for the exchange cross-peaks I_{ab}, I_{ba} were not included as these were absent at the start of the mixing period in all cases. Uncertainties in the fitted parameters were calculated using a Monte-Carlo approach based on the noise of the experiment. Fitting was performed using in-house scripts written in Python employing the LmFit module available at https://lmfit.github.io/lmfit-py/.

The K_D value of 33.6 μM for the native peptide sequence (`Ac-DNEIEVIIVWEKK-NH2`) determined here in 20 mM sodium phosphate, pH 7.4, 150 mM NaCl, 2 mM DTT, 0.02% NaN_3, 5 °C is not directly comparable to the K_D value of 3.7 μM reported in our prior study[1] (for `FAM-peg-DNEIEVIIVWEKK-NH2`) in 20 mM Tris, 150 mM NaCl, 0.01 % Triton-X, pH 7.4 as the two sequences differ and different buffers were used.

^{15}N CPMG relaxation dispersion

^{15}N CPMG relaxation dispersions were recorded at 600 and 750 MHz using a pulse scheme with amide proton decoupling to measure the rates of in-phase ^{15}N coherences.[6] The ^{15}N-CPMG evolution period was set to 40 ms. $^{1}H_N$ constant wave decoupling was applied at a radiofrequency field strength of 11 kHz. The experiment recorded with the relaxation period omitted served as a reference for the calculation of $R_{2,eff}$ rates as a function of ν_{CPMG}. Uncertainties in $R_{2,eff}$ values were obtained from duplicate measurements at two different ν_{CPMG} frequencies. Data were fit to the 2-state model of Scheme 1 using in-house scripts written in Python employing the LmFit module as described previously.[7] To fit CPMG data at temperatures other than 5 °C, where only 600 MHz data are available, the chemical shifts of the bound states were restrained such that they are within 0.2 ppm within their values at 5 °C. This assumption is safe as the chemical shifts of SUMO bound to parent SIM at different temperatures are all within the 0.2 ppm limit.

Ramachandran Calculations

Simulated annealing calculations were carried out in XPLOR-NIH[8] using parameter and topology files that were manually amended to include *N*-methylated amino acids. To generate Ramachandran plots, the ϕ, ψ backbone dihedral angles were varied in a grid search and a simulated annealing calculation was performed at each point of the search to minimize a pseudo-energy potential that included terms to describe violations from ideal bond geometry and van der Waals clashes. Note that this treatment does not include any attractive energy terms and thus the resulting Ramachandran plots are slightly different than the expected.

SIM peptide characterisation by analytical HPLC and mass spectrometry

Analytical HPLC and mass spectrometry characterisation of peptides used in this study is shown below. In all parts (except g and k): left = analytical HPLC trace, centre = raw nanospray mass spectrum collected in positive ion mode, right = deconvoluted spectrum. In g and k: left = analytical HPLC trace, right = MALDI-TOF spectrum. Where not otherwise noted, HPLC was performed at room temperature.

a) Peptide: parent SIM. Sequence: `Ac-DNEIEVIIVWEKK-NH`$_2$. Gradient: 20-80% Buffer B. Expected mass: 1654.88 Da. Observed mass: 1655.04 Da.

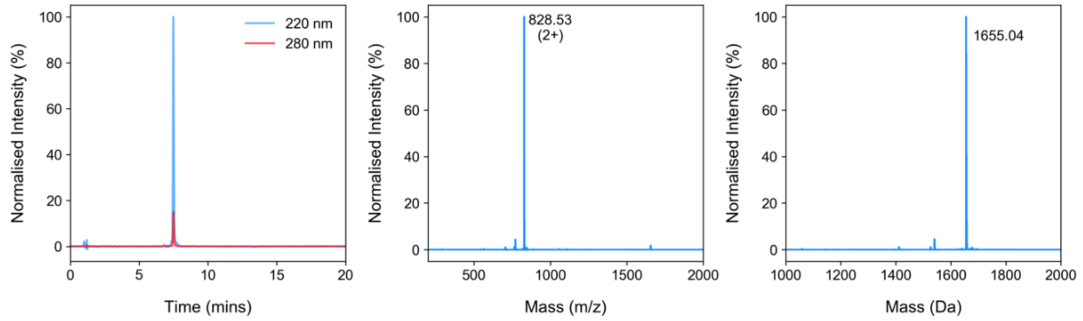

b) Peptide: FAM-SIM. Sequence: `Z-DNEIEVIIVWEKK-NH`$_2$; Z = 5’6 fluorescein–PEG$_2$CH$_2$C(=O). Gradient: 20-80% Buffer B. Expected mass: 2117.32 Da. Observed mass: 2117.18 Da.

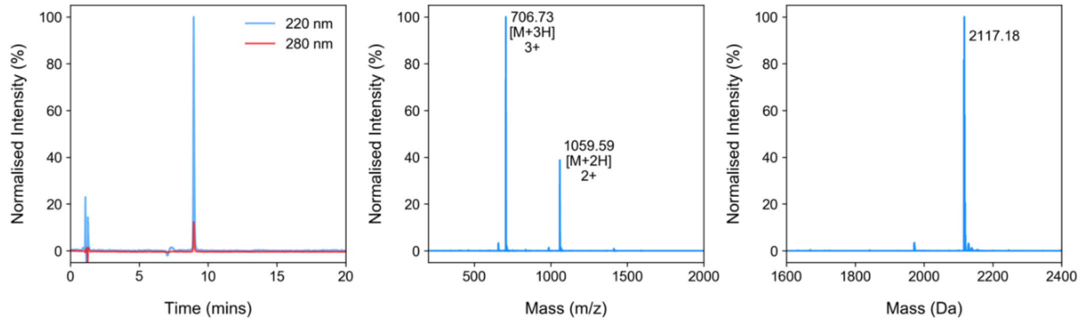

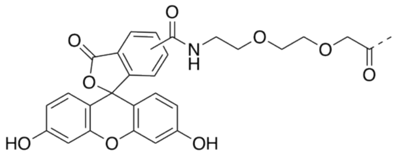

Z = 5’6 fluorescein–PEG$_2$CH$_2$C(=O)

c) Peptide: Me-Asp2705. Sequence: `Ac-(NMe)DNEIEVIIVWEKK-NH`$_2$. Gradient: 20-80% Buffer B. Expected mass: 1668.91 Da. Observed mass: 1669.18 Da.

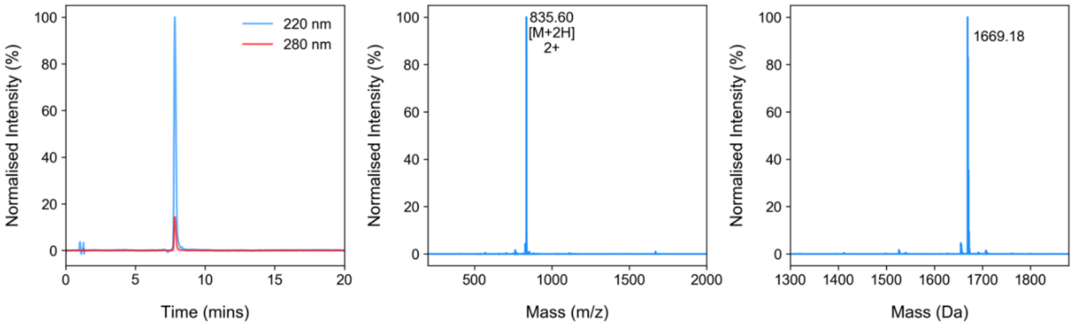

d) Peptide: Me-Asn2706. Sequence: `Ac-D(NMe)NEIEVIIVWEKK-NH`$_2$. Gradient: 20-80% Buffer B. Expected mass: 1668.91 Da. Observed mass: 1668.99 Da.

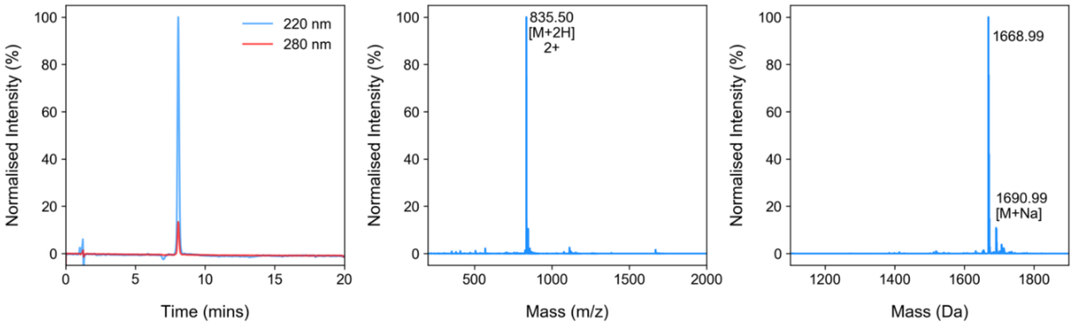

e) Peptide: Me-Glu2707. Sequence: `Ac-DN(NMe)EIEVIIVWEKK-NH`$_2$. Gradient: 20-80% Buffer B. Expected mass: 1668.91 Da. Observed mass: 1669.00 Da.

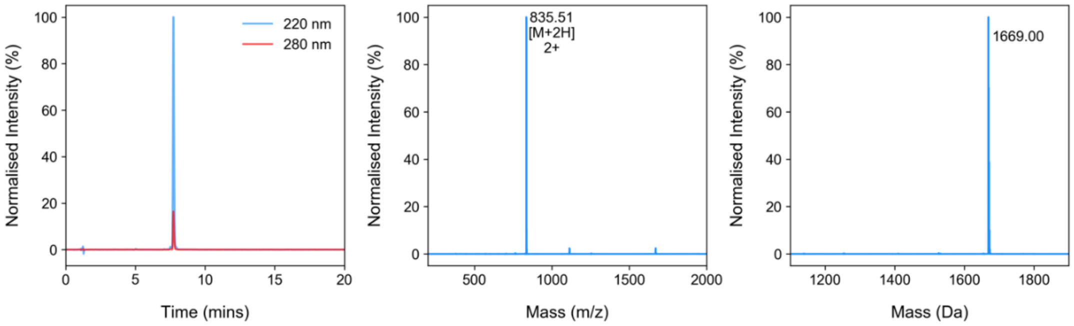

f) Peptide: Me-Ile2708. Sequence: `Ac-DNE(NMe)IEVIIVWEKK-NH`$_2$. Gradient: 20-80% Buffer B. Expected mass: 1668.91 Da. Observed mass: 1668.99 Da.

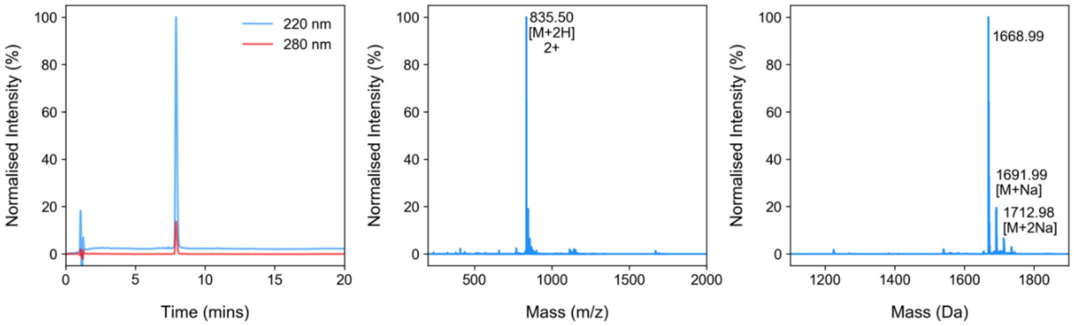

g) Peptide: Me-Glu2709. Sequence: `Ac-DNEI(NMe)EVIIVWEKK-NH`$_2$. Gradient: 20-80% Buffer B. Expected mass (reflector positive mode) = 1669.92 Da. Observed mass: 1670.14 Da.

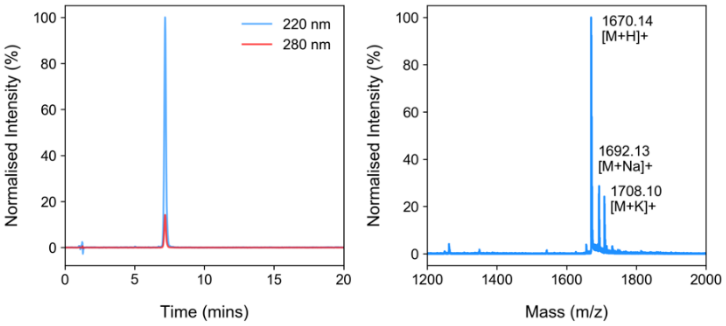

h) Peptide: Me-Val2710. Sequence: Ac-DNEIE(NMe)VIIVWEKK-NH_2. Gradient: 20-80% Buffer B. Expected mass: 1668.91 Da. Observed mass: 1668.59 Da.

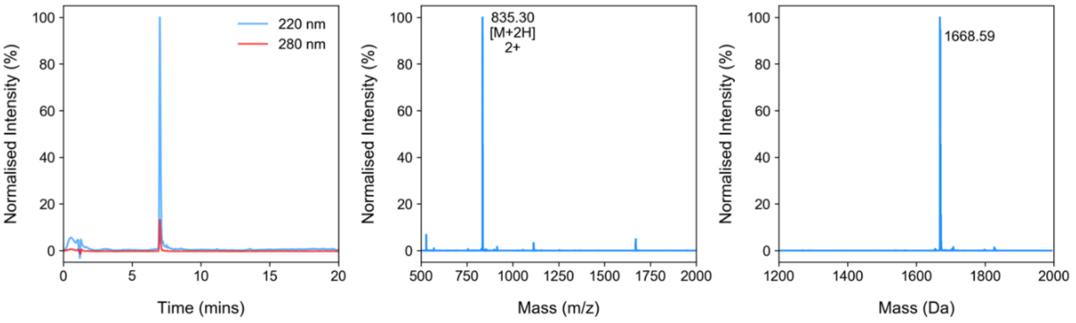

i) Peptide: Me-Ile2711. Sequence: Ac-DNEIEV(NMe)IIVWEKK-NH_2. Gradient: 20-80% Buffer B, 50ºC. Expected mass: 1668.91 Da. Observed mass: 1670.39 Da.

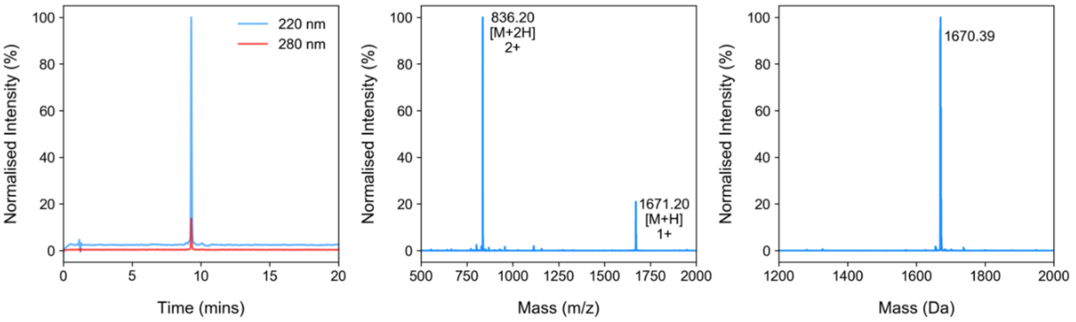

j) Peptide: Me-Ile2712. Sequence: Ac-DNEIEVI(NMe)IVWEKK-NH_2. Gradient: 20-80% Buffer B. Expected mass: 1668.91 Da. Observed mass: 1669.03 Da.

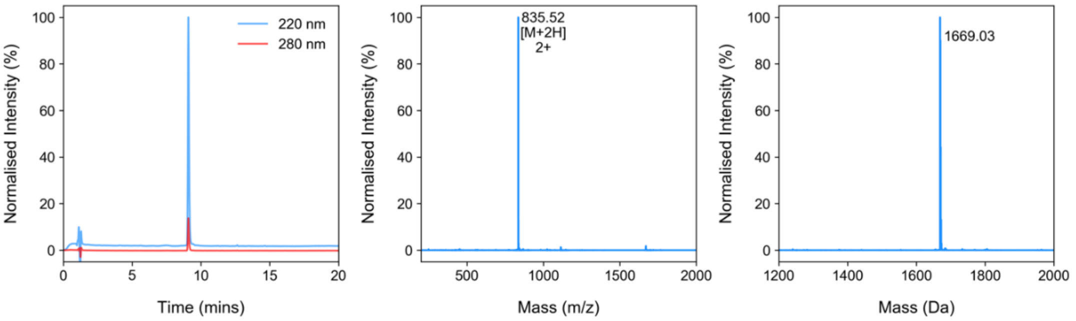

k) Peptide: Me-Val2713. Sequence: Ac-DNEIEVII(NMe)VWEKK-NH_2. Gradient: 20-80% Buffer B. Expected mass (reflector negative mode) = 1668.12 Da. Observed mass: 1668.41 Da.

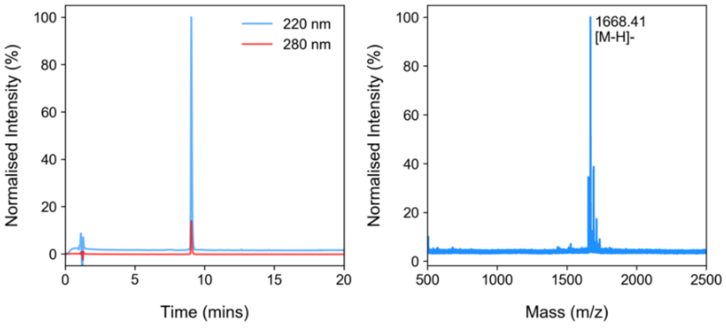

l) Peptide: Me-Trp2714. Sequence: Ac-DNEIEVIIV(NMe)WEKK-NH_2. Gradient: 20-80% Buffer B. Expected mass: 1668.91 Da. Observed mass: 1668.99 Da.

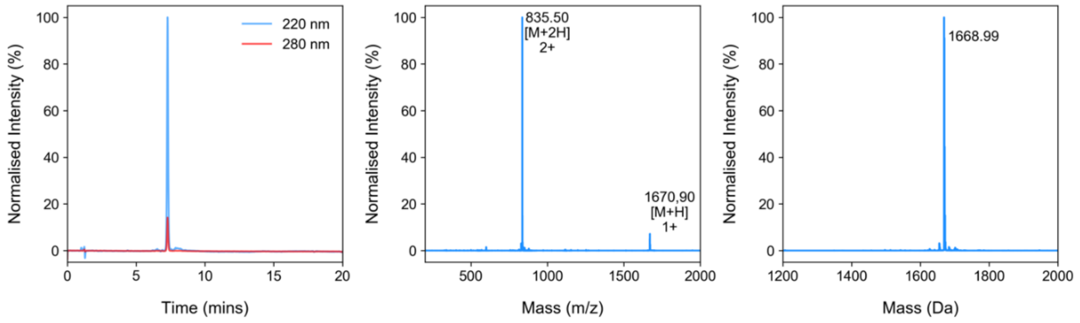

m) Peptide: Me-Glu2715. Sequence: Ac-DNEIEVIIVW(NMe)EKK-NH_2. Gradient: 20-80% Buffer B, 50ºC. Expected mass: 1668.91 Da. Observed mass: 1668.94 Da.

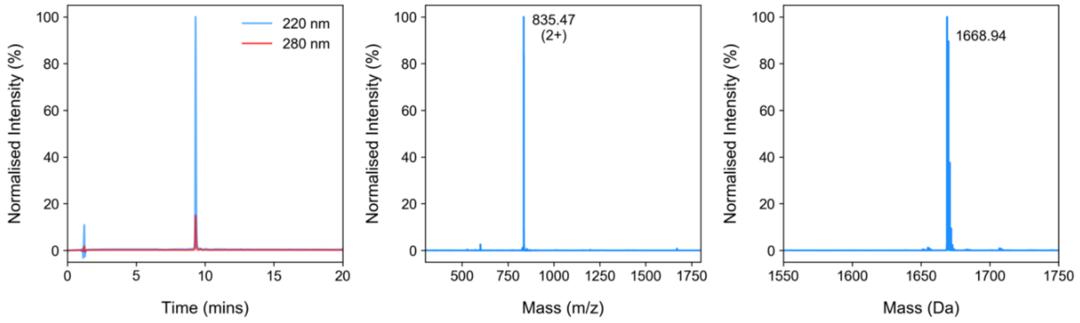

n) Peptide: Me-Lys2716. Sequence: Ac-DNEIEVIIVWE(NMe)KK-NH_2. Gradient: 20-80% Buffer B. Expected mass: 1668.91 Da. Observed mass: 1669.03 Da.

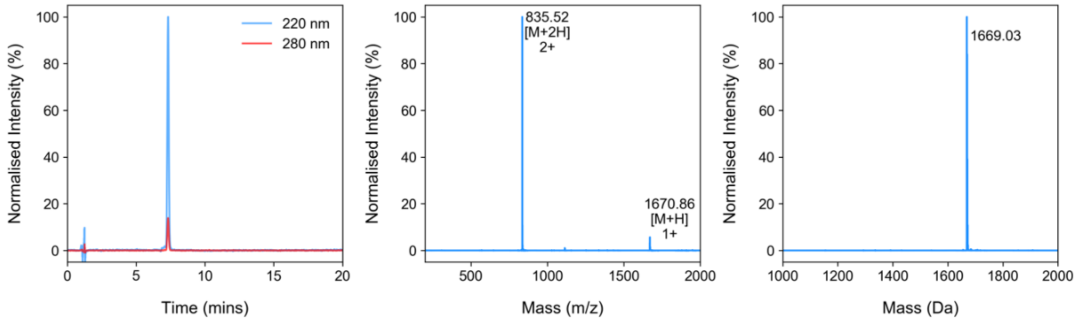

o) Peptide: Me-Lys2717. Sequence: Ac-DNEIEVIIVWEK(NMe)K-NH_2. Gradient: 20-80% Buffer B. Expected mass: 1668.91 Da. Observed mass: 1669.03 Da.

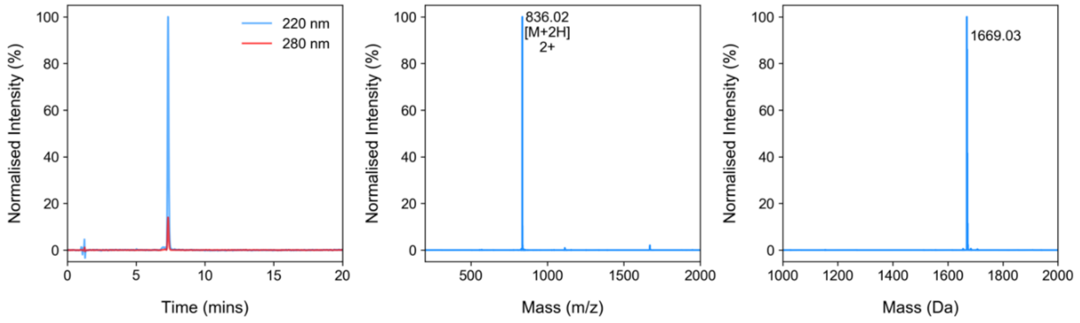

References

1. Ibarra, A. A.; Bartlett, G. J.; Hegedüs, Z.; Dutt, S.; Hobor, F.; Horner, K. A.; Hetherington, K.; Spence, K.; Nelson, A.; Edwards, T. A.; Woolfson, D. N.; Sessions, R. B.; Wilson, A. J., Predicting and Experimentally Validating Hot-Spot Residues at Protein–Protein Interfaces. *ACS Chem. Biol.* **2019,** *14* (10), 2252-2263.
2. The activation barrier for trans/cis isomerization ~66-80 kJ mol-1 (E. Beausoleil, W. D. Lubell, J. Am. Chem. Soc. 1996, 118, 12902-12908) would likely hinder interaction of N-methylated peptides that have the wrong rotamer for binding suggesting that even for the peptides with a detectable cis content this is not mechanistically relevant for binding.
3. Namanja, A. T.; Li, Y.-J.; Su, Y.; Wong, S.; Lu, J.; Colson, L. T.; Wu, C.; Li, S. S. C.; Chen, Y., Insights into High Affinity Small Ubiquitin-like Modifier (SUMO) Recognition by SUMO-interacting Motifs (SIMs) Revealed by a Combination of NMR and Peptide Array Analysis. *J. Biol. Chem.* **2012,** *287* (5), 3231-3240.
4. Farrow, N. A.; Zhang, O.; Forman-Kay, J. D.; Kay, L. E., A heteronuclear correlation experiment for simultaneous determination of 15N longitudinal decay and chemical exchange rates of systems in slow equilibrium. *J. Biomol. NMR* **1994,** *4* (5), 727-734.
5. Kjaergaard, M.; Brander, S.; Poulsen, F. M., Random coil chemical shift for intrinsically disordered proteins: effects of temperature and pH. *J. Biomol. NMR* **2011,** *49* (2), 139-149.
6. Hansen, D. F.; Vallurupalli, P.; Kay, L. E., An Improved 15N Relaxation Dispersion Experiment for the Measurement of Millisecond Time-Scale Dynamics in Proteins. *The Journal of Physical Chemistry B* **2008,** *112* (19), 5898-5904.
7. Karamanos, T. K.; Tugarinov, V.; Clore, G. M., Unraveling the structure and dynamics of the human DNAJB6b chaperone by NMR reveals insights into Hsp40-mediated proteostasis. *Proc. Natl. Acad. Sci. U. S. A.* **2019,** *116* (43), 21529-21538.
8. Schwieters, C. D.; Bermejo, G. A.; Clore, G. M., Xplor-NIH for molecular structure determination from NMR and other data sources. *Protein Sci.* **2018,** *27* (1), 26-40.
